# Parallel mechanochemical synthesis of phenolic drug–poly(lactic acid) conjugates using a multi-tube BenchMixer/vortexer

**DOI:** 10.1039/d6ra07580k

**Published:** 2026-10-26

**Authors:** Mohammad A. Ebqa'ai, Kaleb Cossette, Lydia Park, Mckalay P. Estep, K. D. Priyasantha, Abdullah M. Abu Anzeh, Weinan Tan, Sandun B. K. Dias, Toby L. Nelson

**Affiliations:** a Department of Chemistry, Yarmouk University P.O. BOX 566 Irbid 21163 Jordan mebqaai@yu.edu.jo; b Department of Chemistry, Physics, and Engineering, Cameron University Lawton OK 73505 USA; c Faculty of Science, Coker University Hartsville SC 29550 USA; d Department of Chemistry, University of North Texas Denton TX 76201 USA; e Bredesen Center for Interdisciplinary Research and Graduate Education, The University of Tennessee Knoxville TN 37996 USA; f Oak Ridge Innovation Institute, The University of Tennessee Knoxville TN 37996 USA

## Abstract

A low-cost multi-tube BenchMixer/vortexer platform was developed for solvent-minimized mechanochemical ring-opening polymerization (ROP) of l-lactide to prepare phenolic drug–poly(lactic acid) (PLA) conjugates. The platform can process up to 8 sealed reactions in one run, enabling rapid optimization and substrate screening under identical conditions. Using 4-methoxyphenol as a model initiator, reaction parameters were optimized and then applied to a broad substrate scope including twelve substituted phenols, four bioactive phenolic drugs, and two heteroatom analogues, 4-methoxyaniline and 4-methoxythiophenol. Chlorothymol, acetaminophen, capsaicin, and naloxone were successfully converted into low-molecular-weight drug–PLA conjugates with narrow dispersities. Chlorothymol gave the best overall performance, affording 97% lactide conversion, DP ≈ 36, *M*_*n*_ = 2.68 kDa, *Đ* = 1.16, 6.64% drug loading, and 85% isolated yield. Capsaicin gave the highest drug loading (11.39%), followed by naloxone and acetaminophen. The heteroatom study showed that initiator identity strongly affects polymerization efficiency, following the trend N > O > S. To the best of our knowledge, this is the first systematic use of a low-cost, multi-tube BenchMixer/vortexer platform for phenolic drug-initiated mechanochemical ROP of l-lactide. This parallel, solvent-minimized method enables rapid screening of phenolic and heteroatom initiators and offers a practical greener route to biodegradable drug–PLA conjugates with potential relevance to sustained drug delivery.

## Abstract 2

Parallel BenchMixer-assisted mechanochemistry enables efficient synthesis of phenolic drug–PLA conjugates under low-solvent conditions, highlighting a greener route toward longer-lasting drug delivery systems.

## Graphical abstract

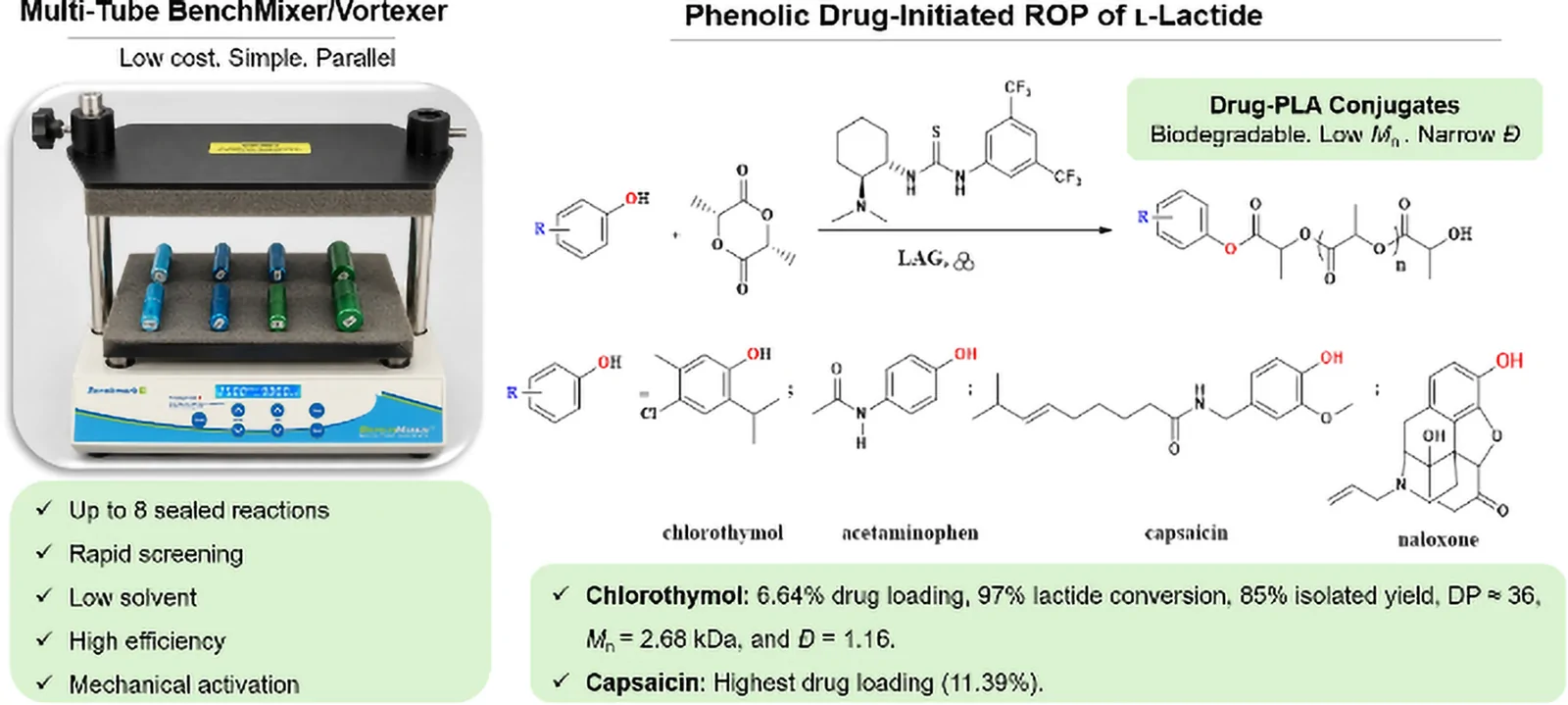

## 1. Introduction

Poly(lactic acid) (PLA) is among the most widely investigated biodegradable polyesters due to its biocompatibility, hydrolytic degradability, and broad utility in biomedical materials. PLA and related aliphatic polyesters have been used in resorbable sutures, tissue-engineering scaffolds, surgical materials, implants, and controlled drug delivery systems.^1,2^ For drug delivery, PLA is particularly attractive because degradation of the polyester backbone can be used to regulate therapeutic release and reduce the need for frequent dosing. Additionally, PLA nanoparticles and drug–polymer conjugates have been investigated to enhance drug stability, bioavailability, and sustained-release characteristics.^3,4^ In this regard, covalent attachment of phenolic drugs to short PLA chains provides a practical method to control the drug loading and release with PLA biodegradability.

Mechanochemistry is a practical approach to achieve more sustainable production of PLA through mechanochemical reactions with minimal or no solvent.^5,6^ This is particularly useful for the synthesis of polymer and drug–polymer as it can decrease the amount of solvents used, simplify the processing, and enable efficient screening of reactions.^7–9^ There are some mechanochemical platforms in which polymer synthesis has been carried out such as shaker ball mill, planetary ball mill, and speed mixer.^7–10^ Shaker mills use the oscillating motion of the milling jar while the planetary mills use the motion of the jar about a central axis as well as the oscillation of the jar itself. Speed-mixers or centrifugal planetary mixers employ dual-axis movement which promotes mixing and energy transfer. These platforms have been successfully applied to lactide polymerization, including ball-milled PLA synthesis, PLA block copolymer formation, phenolic-initiated PLA nanoparticles, and naloxone-initiated PLA synthesis.^4–6,10^

Compared with our previous mechanochemical PLA studies,^4,10^ the present work expands both the platform design and substrate scope. The 2022 study used a shaker ball-milling setup for phenolic-initiated PLA nanoparticle synthesis, while the 2025 study used a speed-mixer approach for naloxone-initiated PLA synthesis. In contrast, the present BenchMixer/vortexer platform is a lower-cost and more accessible setup that can accommodate up to 8 sealed reactions in one run. This platform enabled parallel screening using the same BenchMixer/vortexer system, with several reactions evaluated in a single run. This allowed broader evaluation of substituted phenols, phenolic drugs, and heteroatom initiators, providing a more systematic structure–reactivity comparison for biodegradable drug–PLA conjugate synthesis.

Vortex-based mechanochemistry was previously introduced by Stojaković *et al.* as an automated approach for supramolecular catalysis and metal–organic framework preparation, demonstrating that vortex motion can provide mechanical activation beyond conventional ball milling.^11^ Building on this concept, the present work introduces a simple multi-tube BenchMixer/vortexer as an accessible platform for phenolic drug-initiated ROP of l-lactide. As shown in Fig. 1, the system allows multiple sealed grinding jars to be processed under the same milling conditions, enabling parallel reaction screening.

**Fig. 1:**
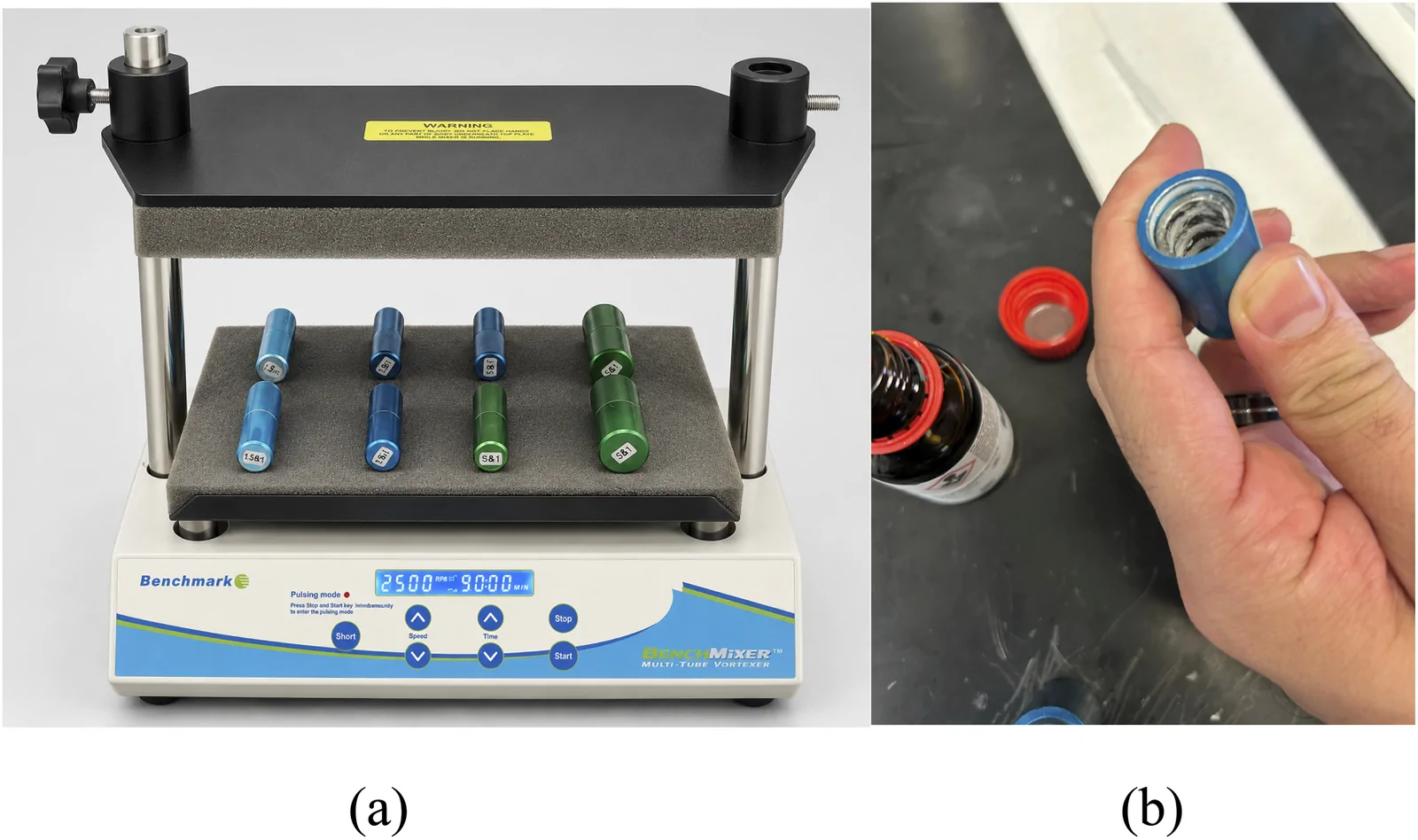
BenchMixer/vortexer-assisted mechanochemistry and proposed ball motion. (a) Multi-tube BenchMixer/vortexer used for parallel reaction screening. (b) Representative ball-motion pattern inside the grinding jar.

The proposed ball-motion pattern inside the cylindrical grinding jar is supported by the circular and arc-shaped abrasion marks observed after milling, which are consistent with repeated rolling, sliding, and impact of the stainless-steel balls during vortex mixing. In this system, back-and-forth oscillation of the mixer and eccentric ball motion inside the jars promote mechanical contact between lactide, initiator, catalyst, and the liquid-assisted grinding agent (LAG). Compared with traditional ball-milling and speed-mixing approaches, this BenchMixer setup is low-cost, easy to operate, and capable of processing multiple reactions simultaneously (Table S1), making it useful for rapid substrate screening. By combining liquid-assisted grinding with a multi-tube BenchMixer/vortexer, this method reduces solvent demand, simplifies reaction setup, and enables parallel screening under identical conditions. To the best of our knowledge, this is the first systematic application of a multi-tube BenchMixer/vortexer for phenolic drug-initiated mechanochemical ROP of l-lactide, providing a practical platform for greener polymer–drug conjugate synthesis.

Phenolic drugs are good candidates for PLA conjugation, as the scaffold of the drug has biological activity and the phenolic O–H group is a reactive handle for the ring-opening polymerization. In this study, the substrate scope included sixteen phenolic initiators: twelve substituted phenols and four bioactive phenolic drugs, namely acetaminophen, naloxone, capsaicin, and 4-chloro-2-isopropyl-5-methylphenol, commonly known as chlorothymol (Scheme 1). To further examine the role of the initiating heteroatom, 4-methoxyaniline and 4-methoxythiophenol were also evaluated alongside 4-methoxyphenol, allowing direct comparison of oxygen-, nitrogen-, and sulfur-based initiation under the same mechanochemical conditions. This substrate design enabled the effects of electronic structure, steric accessibility, and heteroatom identity to be assessed using a single BenchMixer/vortexer platform.

**Scheme 1:**
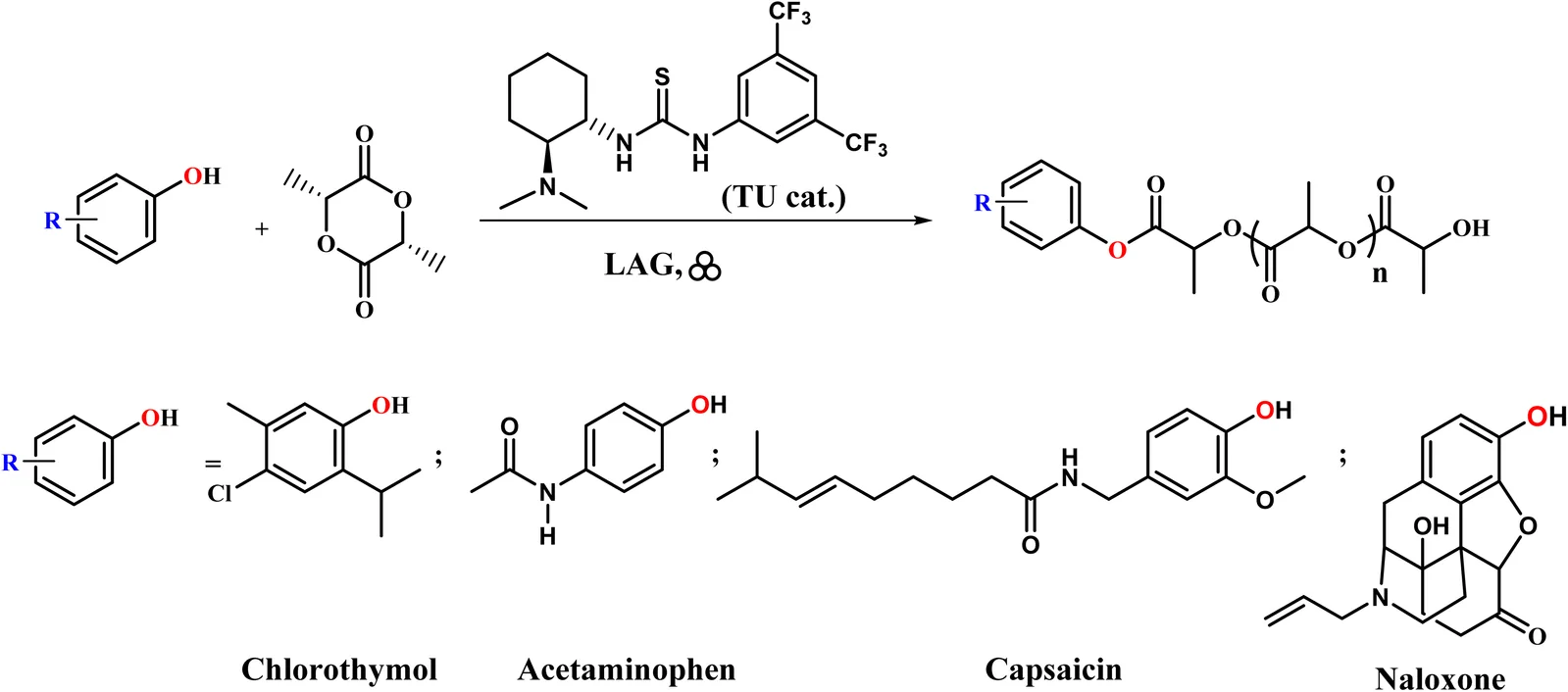
Mechanochemical ROP of l-lactide using phenolic drugs as initiators.

The purpose of covalently attaching phenolic drugs to PLA is to prepare biodegradable drug–polymer conjugates that may support sustained release, reduce rapid clearance, lower dosing frequency, and improve control over drug loading. PLA- and PLGA-based delivery systems commonly rely on physical encapsulation or blending of free drugs with preformed polymer carriers, and these approaches have been widely used. However, they may also face challenges such as burst release, incomplete loading, drug leakage during storage, and less precise control over the amount of drug associated with the carrier.^12–14^ In contrast, drug-initiated ROP provides a covalent polymeric prodrug structure in which the drug is chemically incorporated as the PLA chain end. In this design, the PLA chain length can influence both the relative drug loading and the release profile through hydrolysis or degradation of the polyester backbone. This strategy has been determined for various drug–PLA conjugates, including paclitaxel–PLA, camptothecin–PLA, and naloxone–PLA approaches.^10,15–18^

Acetaminophen is a widely used analgesic and antipyretic drug that has been explored in several drug delivery systems. For example, acetaminophen has been incorporated into PLA, PCL, and related biodegradable polymer matrices for *in vitro* release studies, showing that polymer composition can influence its release behavior.^19^ It has also been investigated for intranasal delivery to the central nervous system using mucoadhesive and absorption-enhancing formulations, further highlighting interest in improving its delivery profile.^20^ These reports support the importance of acetaminophen delivery strategies; however, they mainly rely on formulation or physical loading approaches. In the present work, acetaminophen is used directly as a phenolic initiator for l-lactide polymerization, providing a covalent route to acetaminophen–PLA conjugates.

Naloxone is an opioid receptor antagonist used for emergency opioid-overdose reversal, but its short duration of action has motivated interest in sustained-release delivery systems. Averick and co-workers reported covalently loaded naloxone–PLA nanoparticles as a longer-acting polymer-based formulation.^18^ Related biological studies showed that slow and sustained naloxone delivery from covalent nanoparticles can reduce withdrawal-associated responses compared with free naloxone exposure.^21^ More recently, our group reported naloxone-initiated mechanochemical PLA synthesis as a one-step route to naloxone–PLA materials, further supporting the use of phenolic drugs as initiators for PLA-based delivery systems.^10^

Capsaicin is a phenolic vanillyl amide used mainly in pain-related applications; however, its poor aqueous solubility, irritation, and delivery limitations have encouraged the development of nanoparticle and polymer-based delivery systems. Capsaicin-loaded polymer nanoparticles have been reported for controlled release, and PLGA-based capsaicin formulations have shown the ability to reduce acute itch and heat-pain responses in topical applications.^22–24^ Chlorothymol is another bioactive phenolic compound of interest because chlorinated phenolic structures are widely associated with antimicrobial activity. 4-Chloro-2-isopropyl-5-methylphenol has shown antimicrobial and antibiofilm activity against methicillin-resistant *Staphylococcus aureus*, making it a suitable phenolic antimicrobial scaffold for evaluating PLA conjugation.^25^

Together, these examples show that phenolic drugs are both biologically relevant and chemically suitable for direct incorporation into biodegradable PLA chains. Therefore, the goal of this work is to establish a mechanochemical approach for synthesizing of low-DP phenolic drug–PLA conjugates *via* BenchMixer/vortexer with low amounts of solvent. Polymerizations were conducted at ambient temperature under inert conditions using a thiourea/tertiary amine organocatalytic system and using liquid-assisted grinding agents as well as stainless-steel milling balls. This method offers a viable way to work towards the development of an extended activity phenolic drug delivery systems, and lowers the amount of solvents used, processing time and preparation steps. Although high-molecular-weight PLA is important for functional materials, the present study was designed to target low-DP drug–PLA conjugates using a low lactide-to-initiator ratio. In our previous mechanochemical PLA study, increasing this ratio increased the molecular weight, showing that chain length can be tuned by monomer loading.^4^ Therefore, the BenchMixer/vortexer platform may also be adapted for higher-molecular-weight PLA synthesis by adjusting the lactide-to-initiator ratio, which will be investigated in future work.

## 2. Results and discussion

### 2.1. Impact of liquid-assisted grinding and catalyst loading on phenolic-initiated ROP of l-lactide

Optimization of the reaction conditions was done first using 4-methoxyphenol as a model phenolic initiator before evaluating the broader substrate scope. The optimization process started with two factors: the amount of CHCl_3_ used as a liquid-assisted grinding agent and thiourea/tertiary amine as catalyst. These two variables were chosen first because they directly influence the mixing of reagents, local mobility, contact of the catalyst to the substrate and activation under mechanochemical conditions.^4,7–9^

A representative purified ^1^H NMR spectrum of the 4-methoxyphenol–PLA product obtained from Table 1, entry 4 is shown in Fig. S1. The PLA methine protons appeared at 4.32–5.35 ppm, while the corresponding methyl protons were observed at 1.47–1.67 ppm. The aromatic protons of the 4-methoxyphenol end group appeared at 6.86–7.00 ppm, and the methoxy signal was observed at 3.78 ppm. A hydroxyl proton was also observed at 2.69 ppm. These assignments are consistent with our previous report on 4-methoxyphenol-initiated PLA synthesized under mechanochemical conditions.^4^

**Table 1:** Impact of solvent and catalyst loadings on phenolic-initiated ROP of l-lactide^a^

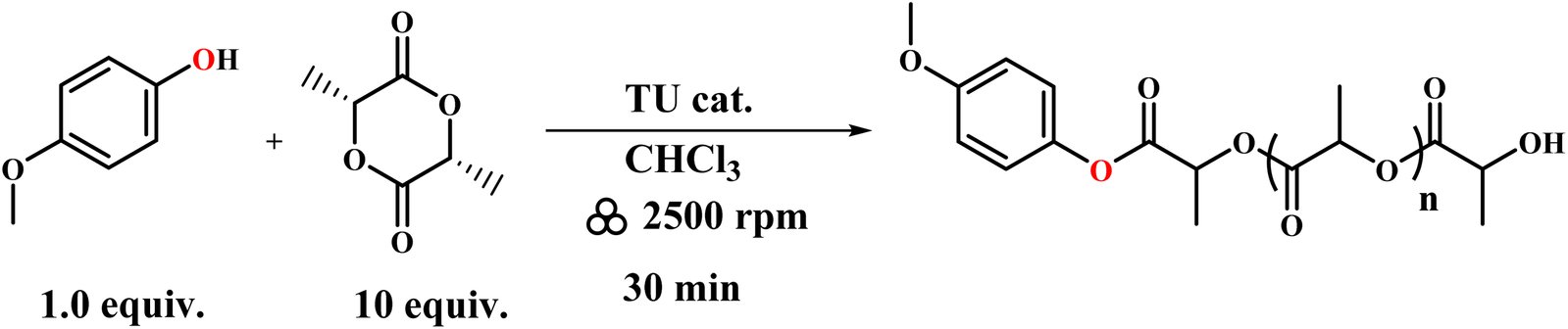						
Entry	TU (equiv.)	CHCl_3_ (µL)	IE^b^ (%)	Conv.^b^ (%)	DP^b^	Yield^c^ (%)
1	1.25	0	NR	NR	NR	NR
2	1.25	10	58	57	24	32
3	1.25	20	69	69	27	41
4	1.25	30	65	79	30	46
5	1.25	40	75	91	30	67
6	1.25	50	81	84	47	62
7	0.50	40	65	71	30	42
8	1.00	40	67	85	30	59
9	1.50	40	80	99	46	82

a Polymerization conditions: l-lactide (100 mg, 0.694 mmol, 10 equiv.), 4-methoxyphenol (8.60 mg, 0.0694 mmol, 1 equiv.) in a 5 mL stainless-steel jar with 5 mm (×4) stainless-steel balls for 30 min at 2500 rpm speed.

b Determined *via*^1^H NMR.

c Isolated yield.

The degree of polymerization (DP) was determined by ^1^H NMR end-group analysis using the integration ratio between the phenolic end-group proton and the PLA methine proton from the polymer chain (eqn (S1)). Lactide conversion was calculated from the crude ^1^H NMR spectra by comparing the methyl resonances of PLA and residual l-lactide (eqn (S2)). Initiation efficiency (IE%) was determined from the relative integrations of polymer-bound phenolic initiator and unreacted free phenol in the crude ^1^H NMR spectra (eqn (S3)). Thus, ^1^H NMR provided a direct method to evaluate monomer conversion, initiator incorporation, and average PLA chain length.

The influence of LAG amount was initially investigated by increasing the amount of CHCl_3_ in the range of 0–50 µL while keeping the loading of the catalyst at 1.25 equiv. No polymerization was observed in the absence of CHCl_3_, suggesting that neat grinding alone, under these conditions, was not enough to promote the process of the ROP of l-lactide with the phenolic initiator. By simply adding 10 µL of CHCl_3_, polymer formation was achieved with 58% initiation efficiency and 57% lactide conversion (Table 1, entry 2). The reaction was significantly improved by increasing the volume of CHCl_3_ to 40 µL, while maintaining the other conditions constant, and the 75% initiation efficiency, 91% conversion, DP ≈ 31, and 67% isolated yield were achieved (Table 1, entry 5). This improvement indicates that some liquid is beneficial for the mobility of the reagents, mass transfer and contact between the growing polymer chains and the phenolic initiator with catalyst, which is consistent to what was reported in mechanochemical lactide polymerization.^4–6^

When the volume of CHCl_3_ was further increased to 50 µL, the initiation efficiency was found to be 81% but it resulted in a significantly higher DP of ∼47 and only a moderate improvement in the isolated yield (Table 1, entry 6). This condition was beneficial to the incorporation of the initiator, but the longer PLA chains were found which is not desirable for drug–PLA conjugates, as it leads to a decrease in the relative drug content of the final product. Therefore, 40 µL of CHCl_3_ was chosen as a suitable amount of LAG, which yielded the optimum value for the conversion efficiency, initiation efficiency, DP control and isolated yield.

The loading of the catalyst also was found to significantly influence the polymerization performance. With 0.50 equiv. of the catalyst, the initiation efficiency, lactide conversion and the isolated yield decreased, indicating that the catalyst activation under these conditions was not sufficient (Table 1, entry 7). With an increased loading of 1.00 equiv. the conversion and yield increased but the initiation efficiency remained almost the same (Table 1, entry 8). Increased the catalyst loading to be 1.5 equiv. led to increase the DP (∼46), initiation efficiency, conversion and isolated yield (Table 1, entry 9). This behavior is consistent with thiourea-based organocatalytic ROP, where hydrogen-bonding activation of lactide and the initiating or propagating chain end can accelerate both ring opening and chain growth.^27–29^

Based on this first optimization, 1.25 equiv. catalyst and 40 µL CHCl_3_ were selected as the preferred CHCl_3_-assisted conditions for the next stage of optimization (Table 1, entry 5). Although higher catalyst loading improved conversion and yield, it also increased chain length, which is not ideal for preparing low-DP drug–PLA conjugates with higher relative drug content. Importantly, this optimized system does not require K_2_CO_3_, which was previously shown to increase DP and reduce initiation efficiency in phenolic-initiated mechanochemical PLA synthesis.^4^ Overall, these results show that careful control of both LAG amount and catalyst loading is essential for balancing polymerization efficiency, initiator incorporation, and molecular weight control in BenchMixer-assisted ROP of l-lactide.

### 2.2. Impact of the milling time and milling frequency on the phenols-initiated ROP of l-lactide

After optimizing the LAG amount and catalyst loading, the effects of milling time and milling frequency were examined using 4-methoxyphenol as the model initiator (Table 2). These parameters are especially important in mechanochemical polymerization because they control the energy input, mixing efficiency, reagent contact, and local mobility within the milling jar.^7–9^

**Table 2:** Impact of milling time and milling frequency on phenolic-initiated ROP of l-lactide^a^

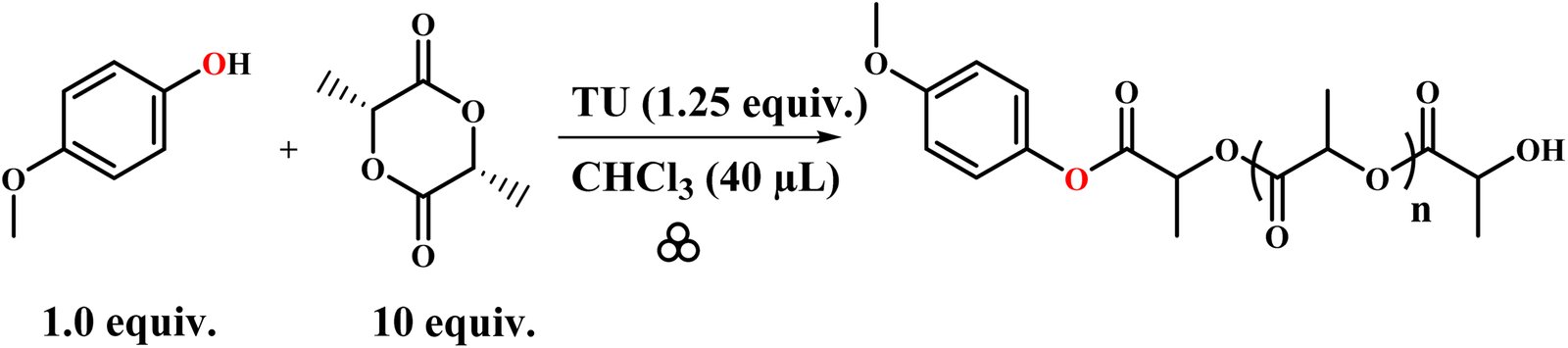						
Entry	Milling frequency (rpm)	Time (min)	IE^b^ (%)	Conv.^b^ (%)	DP^b^	Yield^c^ (%)
1	2500	30	75	91	30	67
2	2500	60	76	95	31	69
3	2500	90	76	>99	32	82
4	2000	90	55	37	27	11
5	1500	90	41	35	26	9

a Polymerization conditions: l-lactide (100 mg, 0.694 mmol, 10 equiv.), 4-methoxyphenol (8.60 mg, 0.0694 mmol, 1 equiv.) in a 5 mL stainless-steel jar with 5 mm (×4) stainless-steel balls.

b Determined *via*^1^H NMR.

c Isolated yield.

The results presented in Table 2 indicate that the lactide conversion and isolated yield were mainly improved with the increase in milling time from 30 to 90 min, whereas the initiation efficiency and DP were nearly unaffected. At 30 min, the reaction gave 75% IE, 91% conversion, DP ≈ 31, and 67% yield (Table 2, entry 1). The milling time was increased to 60 min, which led to a conversion of 95% and yield of 69%, while only a slight increase in IE and DP was observed (Table 2, entry 2). It was found that almost complete conversion was obtained after 90 min, resulting in >99% conversion and 82% yield, whereas IE and DP were around 76% and 32%, respectively (Table 2, entry 3). This behavior suggests that most effective initiation likely occurred during the early stage of milling, establishing a similar number of growing PLA chains, consistent with the nearly constant IE (75–76%) between 30 and 90 min. Because the lactide-to-initiator ratio was fixed, extending the milling time mainly promoted further consumption of residual lactide, as reflected by the increase in conversion from 91% to >99%. Accordingly, the calculated DP showed a modest progressive increase from 30.06 at 30 min to 31.25 at 60 min and 32.47 at 90 min, accompanied by improved product recovery.

Compared to milling time, milling frequency had a greater impact on the ROP results. When the frequency was reduced from 2500 to 2000 rpm at 90 min, initiation efficiency, lactide conversion, and isolated yield decreased sharply. At 2000 rpm, the reaction gave only 55% IE, 37% conversion, and 11% yield (Table 2, entry 4). A further decrease to 1500 rpm gave 41% IE, 35% conversion, and 9% yield (Table 2, entry 5). These results suggest that lower milling frequencies do not provide sufficient mechanical energy, mixing efficiency, or reagent contact to promote effective ROP of l-lactide with the phenolic initiator, catalyst, and LAG agent. This behavior is consistent with previous mechanochemical polymerization studies, where energy input and mixing efficiency were shown to strongly affect polymer formation and conversion.^4,6,30^ Based on these results, 2500 rpm for 90 min was selected as the optimized milling condition for subsequent substrate-scope studies. The reaction was not extended beyond 90 min because nearly complete conversion had already been achieved under these conditions. Overall, this optimization confirms that sufficient milling energy is required to obtain high conversion, good isolated yield, stable initiation efficiency, and controlled DP in phenolic-initiated mechanochemical ROP of l-lactide.

### 2.3. Impact of screening different liquid-assisted grinding agents on phenolic-initiated ROP of l-lactide

After optimizing catalyst loading, LAG amount, milling time, and milling frequency, different liquid-assisted grinding agents were screened to evaluate how the liquid additive affects phenolic-initiated ROP of l-lactide. This parameter was examined because LAG agents can influence reagent wetting, local mobility, substrate–catalyst contact, mixture consistency, and mechanical energy transfer inside the milling jar.^7,8,31^

As shown in Table 3, CHCl_3_ was an effective LAG agent, giving 76% IE, >99% conversion, DP ≈ 32, and 82% yield. THF also gave high conversion (97%), but with lower IE (60%) and a slightly higher DP (≈36). Toluene afforded >99% conversion, 71% IE, DP ≈ 34, and 80% yield. Interestingly, hexane, the least polar LAG agent tested, gave the highest IE (83%) and the lowest DP (≈26), although the conversion decreased slightly to 93%. Overall, these results suggest that less polar LAG agents favor initiator incorporation and lower chain growth, while more polar LAG agents promote higher lactide conversion and longer PLA chains.

**Table 3:** Screen different LAG agents on phenolic-initiated ROP of l-lactide^a^

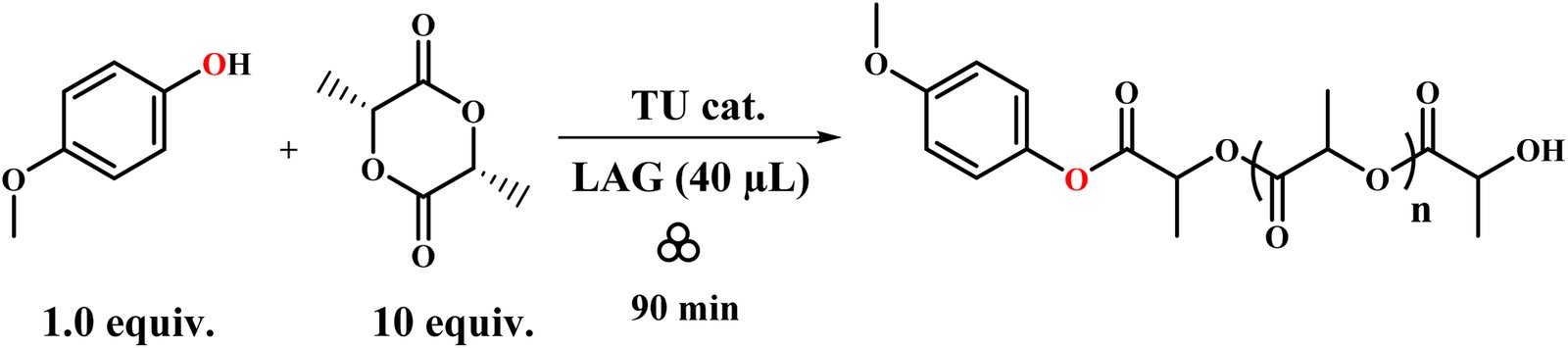							
Entry	TU (equiv.)	LAG	Dielectric constant (*ε*)	IE^b^ (%)	Conv.^b^ (%)	DP^b^	Yield^c^ (%)
1	1.25	CHCl_3_	4.8	76	>99	32	82
2	1.25	THF	7.6	60	97	36	75
3	1.25	Toluene	2.4	71	>99	34	80
4	1.25	Hexane	1.89	83	93	26	74
5	1.00	Hexane	1.89	81	81	32	43
6	1.50	Hexane	1.89	84	>99	25	80

a Polymerization conditions: l-lactide (100 mg, 0.694 mmol, 10 equiv.), 4-methoxyphenol (8.60 mg, 0.0694 mmol, 1 equiv.) in a 5 mL stainless-steel jar with 5 mm (×4) stainless-steel balls at 2500 rpm speed.

b Determined *via*^1^H NMR.

c Isolated yield.

These results are in line with our previous research on phenolic-initiated mechanochemical PLA synthesis, where conversion, initiation efficiency, DP, and yield were all significantly impacted by the LAG agent.^4^ Here, a similar pattern was noted: in comparison to less polar LAG agents, THF produced good conversion but reduced IE and increased DP. Variations in the dielectric constant and solvent–substrate interactions might be connected to this behavior. On the other hand, hexane has a low dielectric constant, which may prevent long-term chain formation while increasing the physical contact between the catalyst, lactide, and phenolic initiator and decreasing the ability to solvate polar propagating compounds.^32–34^

The lower DP obtained with hexane may also reflect differences in mixture flow and force transfer during milling. In mechanochemical systems, small amounts of liquid can change lubrication, collision efficiency, and particle contact. Hexane may provide enough liquid to improve contact between the initiator, lactide, and catalyst, but not enough solvation or local mobility to support extended chain growth. This explanation agrees with broader mechanochemical studies showing that reaction outcomes are highly sensitive to the balance between impact, shear, surface contact, and energy transfer.^35^

Because hexane gave the highest initiation efficiency and the lowest DP among the LAG agents tested, catalyst loading was further examined under hexane-assisted conditions. This experiment complemented the earlier catalyst-loading study carried out with CHCl_3_ and aimed to improve conversion while maintaining low chain length. With 1.00 equiv. catalyst, IE remained high at 81%, but conversion and isolated yield decreased to 81% and 43%, respectively, with DP ≈ 32 (Table 3, entry 5). Increasing the catalyst loading to 1.50 equiv. improved the reaction, giving 84% IE, >99% conversion, DP ≈ 25, and 80% isolated yield (Table 3, entry 6). Therefore, Table 3, entry 6 was selected as the final optimized condition for the substrate-scope studies. The optimized hexane-assisted condition provided high conversion, high initiation efficiency, low DP, and good isolated yield using only a small amount of liquid additive, demonstrating an efficient solvent-minimized route to low-molecular-weight PLA conjugates.

Interestingly, this trend differed from the CHCl_3_ assisted system, where increasing the catalyst loading improved conversion but also increased DP. Under hexane-assisted conditions, higher catalyst loading improved conversion while maintaining a shorter PLA chain length. Overall, the LAG screening shows that nonpolar LAG conditions can provide a better balance between initiator incorporation and molecular weight control, making hexane a suitable liquid additive for preparing low-DP phenolic drug–PLA conjugates.

The preliminary optimization experiments were primarily designed to identify reaction conditions that afforded high monomer conversion and initiation efficiency (IE), together with relatively low and controlled DP, and were therefore evaluated mainly by ^1^H NMR spectroscopy. GPC analysis was subsequently performed for selected polymers prepared under the optimized conditions, and all available molecular weight and dispersity (*Đ*) values are reported herein.

### 2.4. Scope of synthesis with different phenolic initiators

After establishing the optimized hexane-assisted conditions, the substrate scope was evaluated using a series of substituted phenolic initiators (Scheme 2 and Table 4). Methoxy- and alkoxy-substituted phenols were first examined to understand how substituent position and steric effects influence phenolic-initiated ROP of l-lactide. *Para*-substituted phenols gave the best overall balance between initiation efficiency, conversion, DP, and isolated yield. For example, 4-methoxyphenol afforded 84% IE, >99% conversion, DP ≈ 25, and 80% yield, while 4-butoxyphenol gave 75% IE, >99% conversion, DP ≈ 26, and 72% yield. According to these findings, *para*-alkoxy substituents offer advantageous electronic donation while maintaining the phenolic O–H group's accessibility for catalyst-assisted initiation. More growing chains are produced by efficient initiation, which helps maintain lower DP by distributing lactide consumption over more chains.^27–29^

**Scheme 2:**
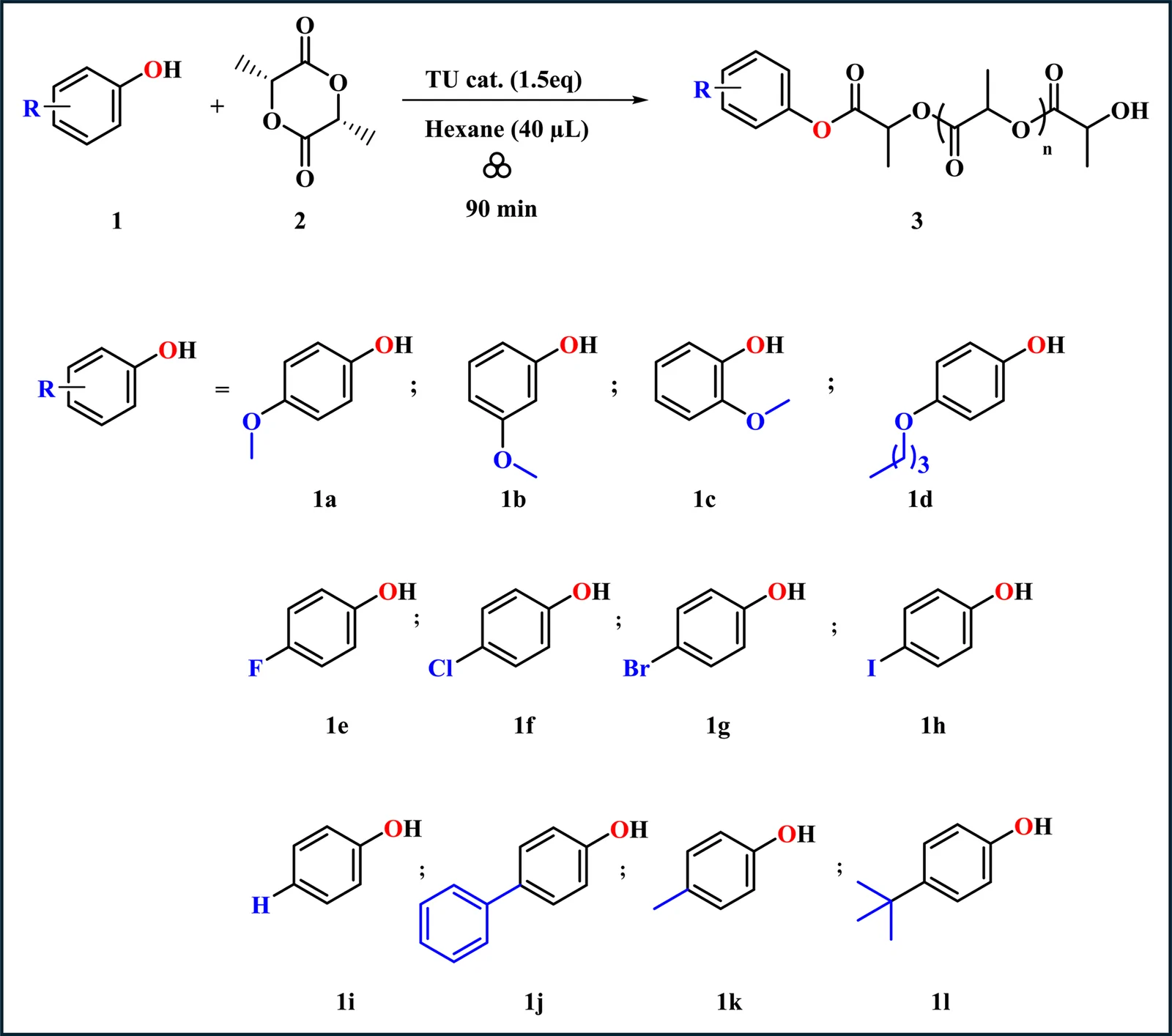
Substate scope of different phenolic initiators.

**Table 4:** Substrate scope of different phenolic initiators^a^

Entry	1	Initiator name	IE^b^ (%)	Conv.^b^ (%)	DP^b^	Yield^c^ (%)
1	1a	*p*-Methoxyphenol	84	>99	25	80
2	1b	*m*-Methoxyphenol	34	94	39	61
3	1c	*o*-Methoxyphenol	37	>99	47	76
4	1d	*p*-Butoxyphenol	75	>99	26	72
5	1e	*p*-Fluorophenol	62	>99	39	75
6	1f	*p*-Chlorophenol	55	>99	39	83
7	1g	*p*-Bromophenol	55	96	45	66
8	1h	*p*-Iodophenol	53	80	43	41
9	1i	Phenol	40	78	35	63
10	1j	*p*-Phenyl-phenol	48	72	40	64
11	1k	*p*-Methylphenol	50	90	29	67
12	1l	*p-Tert*-butylphenol	55	>99	28	78

a Polymerization conditions: l-lactide (100 mg, 0.694 mmol, 10 equiv.), initiator (0.0694 mmol, 1.0 equiv.) in a 5 mL stainless-steel jar with 5 mm (×4) stainless-steel balls at 2500 rpm speed.

b Determined *via*^1^H NMR (S5–S16).

c Isolated yield.

In contrast, *meta*- and *ortho*-methoxy isomers had reduced initiation efficiency and increased DP. *m*-Methoxyphenol yielded only 34% IE, 94% conversion, and DP ≈ 39, indicating that the *meta*-methoxy group offers less electrical support to the phenolic oxygen than the *para* isomer.^36–38^*o*-Methoxyphenol had low IE (37%) despite >99% conversion, and the highest DP in the series (≈47). This finding is most likely due to steric hindrance at the phenolic O–H group and probable intramolecular hydrogen bonding, both of which might reduce the efficient of initiator activation. As a result, fewer initiator molecules participate in chain initiation, leading to longer PLA chains and higher DP.^39,40^ Overall, *para* substitution appears most favorable because it combines beneficial electronic effects with minimal steric interference.

The *para*-halophenol initiators were then examined to evaluate the effect of electron-withdrawing substituents on phenolic-initiated ROP of l-lactide (Table 4, entries 5–8). Among this series, 4-fluorophenol gave the best performance, affording 62% IE, >99% conversion, DP ≈ 39, and 75% yield. 4-Chlorophenol also reached >99% conversion, but with slightly lower IE (55%) and a similar DP (≈39), giving 83% yield. 4-Bromophenol showed comparable IE (55%) but lower conversion (96%) and a higher DP (≈45). In contrast, 4-iodophenol gave the weakest overall result, with 53% IE, 80% conversion, DP ≈ 43, and 41% yield. Overall, the halophenols showed moderate initiation efficiency and generally higher DP compared with the *para*-alkoxy phenols.

This trend can be explained by the balance between the inductive and resonance effects of the halogen substituent. All halogens withdraw electron density through the inductive effect, but fluorine can partially compensate through resonance donation because its compact 2p orbitals overlap effectively with the aromatic π-system. This may help 4-fluorophenol maintain greater electron density at the phenolic oxygen after activation, supporting more efficient initiation. In contrast, chlorine, bromine, and iodine have larger and more diffuse orbitals that overlap less effectively with the aromatic ring, resulting in weaker resonance donation and less favorable initiation. This explains why the heavier halophenols showed lower initiation efficiency and higher DP under the optimized mechanochemical conditions.^36,38,41–43^

These results are consistent with our previous phenolic-initiated mechanochemical PLA study, where *p*-iodophenol showed lower initiation efficiency and higher DP than *p*-methoxyphenol, mainly due to the reduced nucleophilicity of the phenolic oxygen caused by the electron-withdrawing iodo substituent.^4^ In the present work, the halophenols again showed lower IE and higher DP than the *para*-alkoxy phenols, supporting the conclusion that electron-donating substituents favor initiator incorporation, whereas electron-withdrawing halogens reduce initiation efficiency. Overall, 4-fluorophenol was the most effective halophenol initiator, while the heavier halogens, especially iodine, were less favorable under the optimized BenchMixer/vortexer conditions.

The parent phenol and *para*-hydrocarbon-substituted phenols were then evaluated to examine how non-heteroatom electron-donating groups affect phenolic-initiated ROP of l-lactide (Table 4, entries 9–12). Phenol gave moderate performance, with 40% IE, 78% conversion, DP ≈ 35, and 63% yield. Introducing a *para*-methyl group improved the reaction, giving 50% IE, 90% conversion, DP ≈ 29, and 67% yield. The best result in this series was obtained with *p-tert*-butylphenol, which afforded 55% IE, >99% conversion, DP ≈ 28, and 78% yield. This trend suggests that alkyl substituents can enhance initiation by increasing electron density at the phenolic oxygen through inductive donation. More efficient initiation produces a larger number of growing chains, helping reduce the average DP.^36,38,41^

The result obtained with *p-tert*-butylphenol is particularly interesting. Although the *tert*-butyl group is bulky, it is located at the *para* position and therefore does not strongly interfere with activation of the phenolic O–H group. At the same time, its alkyl character can increase electron donation to the phenolic oxygen. The hydrophobic nature of this substituent may also make the initiator more compatible with the nonpolar hexane-assisted medium, which could improve mixing and contribute to the high conversion observed. By comparison, *p*-phenylphenol was less effective, giving 48% IE, 72% conversion, DP ≈ 40, and 64% yield. This lower performance may reflect the larger and more rigid phenyl group, which can limit mixing or substrate mobility during milling. The phenyl substituent is also a weaker electron donor than alkyl groups, which may explain its lower ability to promote efficient phenolic initiation.^44^

Fig. 2 summarizes the substrate-scope results by comparing initiation efficiency, lactide conversion, and DP for the phenolic initiators. Overall, *para*-alkoxy phenols, especially *p*-methoxyphenol and *p*-butoxyphenol, provided the best balance of high IE, near-complete conversion, and low DP. In contrast, the *meta*- and *ortho*-methoxy isomers showed lower IE and higher DP, while the halophenol series gave moderate IE with generally longer PLA chains. The hydrocarbon-substituted phenols showed improved performance compared with phenol, particularly *p-tert*-butylphenol, which gave high conversion and low DP. These trends confirm that both electronic effects and steric accessibility of the phenolic O–H group strongly influence initiator performance in BenchMixer/vortexer-assisted ROP of l-lactide.

**Fig. 2:**
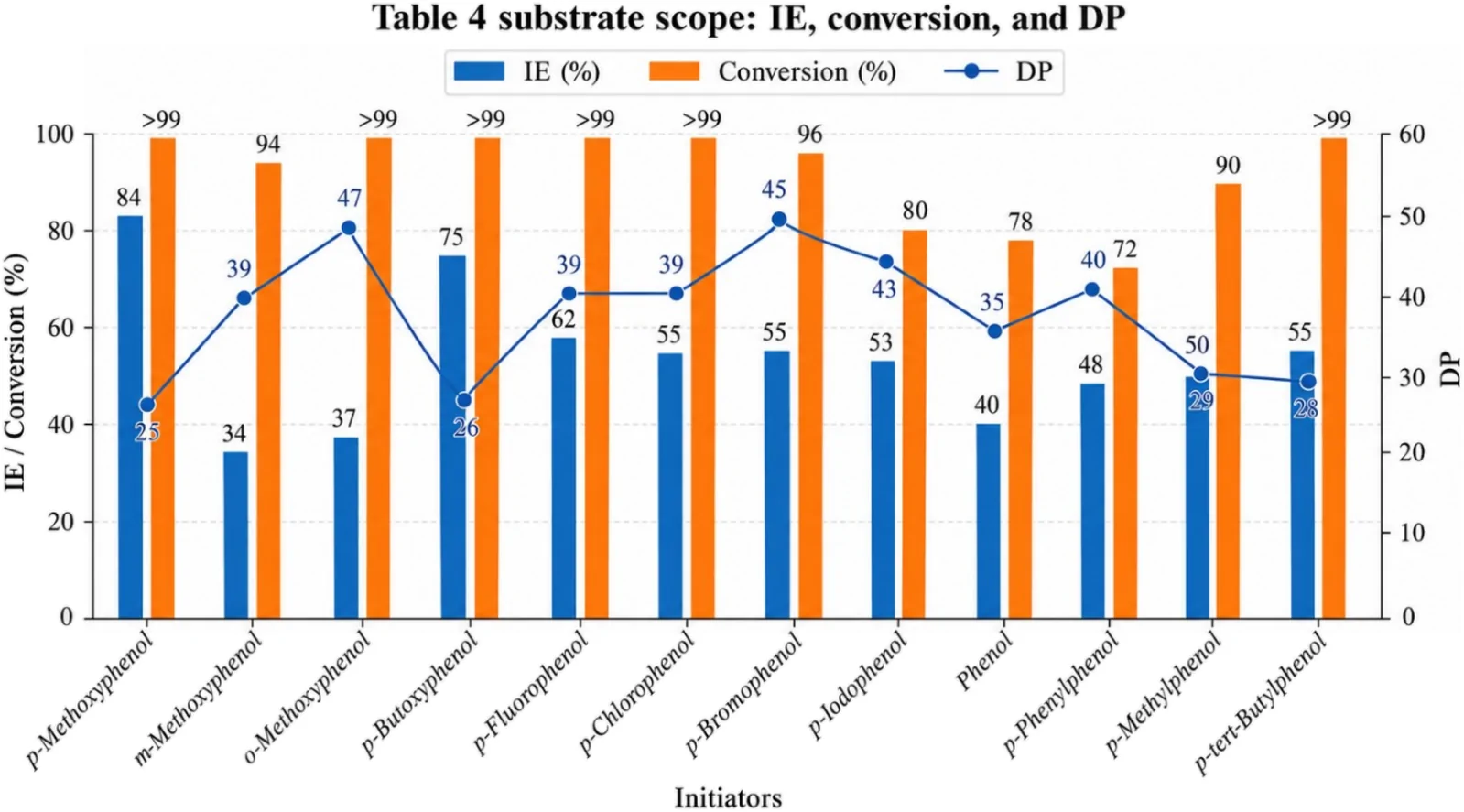
Effect of phenolic initiator structure on initiation efficiency, conversion, and DP in bench mixer-assisted ROP of l-lactide.

Importantly, the present BenchMixer/vortexer method improved DP control compared with our previous phenolic-initiated mechanochemical PLA synthesis using ball milling.^4^ In the previous method, phenol, *p*-methoxyphenol, and *p*-iodophenol gave DP values of 47, 31, and 66, respectively. In the current BenchMixer/vortexer system, lower DP values were obtained for the same or related initiators. This is beneficial for drug–PLA conjugates, because shorter PLA chains increase the relative drug or initiator content in the final material.^10,18^ Overall, *para* electron-donating substituents favored initiation and conversion. They also helped maintain lower DP. In contrast, electron-withdrawing, sterically demanding, or less mobile substrates showed lower initiation efficiency under the optimized BenchMixer/vortexer conditions.

### 2.5. Scope of synthesis with different phenolic-drug initiators

The optimized BenchMixer/vortexer conditions were next applied to four phenolic drug initiators: chlorothymol, acetaminophen, capsaicin, and naloxone (Scheme 3 and Table 5). This part of the study is important because these substrates are more structurally complex than the model phenols. They contain additional functional groups that can influence initiation, propagation, hydrogen bonding, and mixing under mechanochemical conditions. The successful formation of drug–PLA conjugates from these initiators supports the value of this low-solvent method for preparing covalently linked biodegradable polymer–drug materials with potential relevance to sustained drug delivery.^4,10,18^

**Scheme 3:**
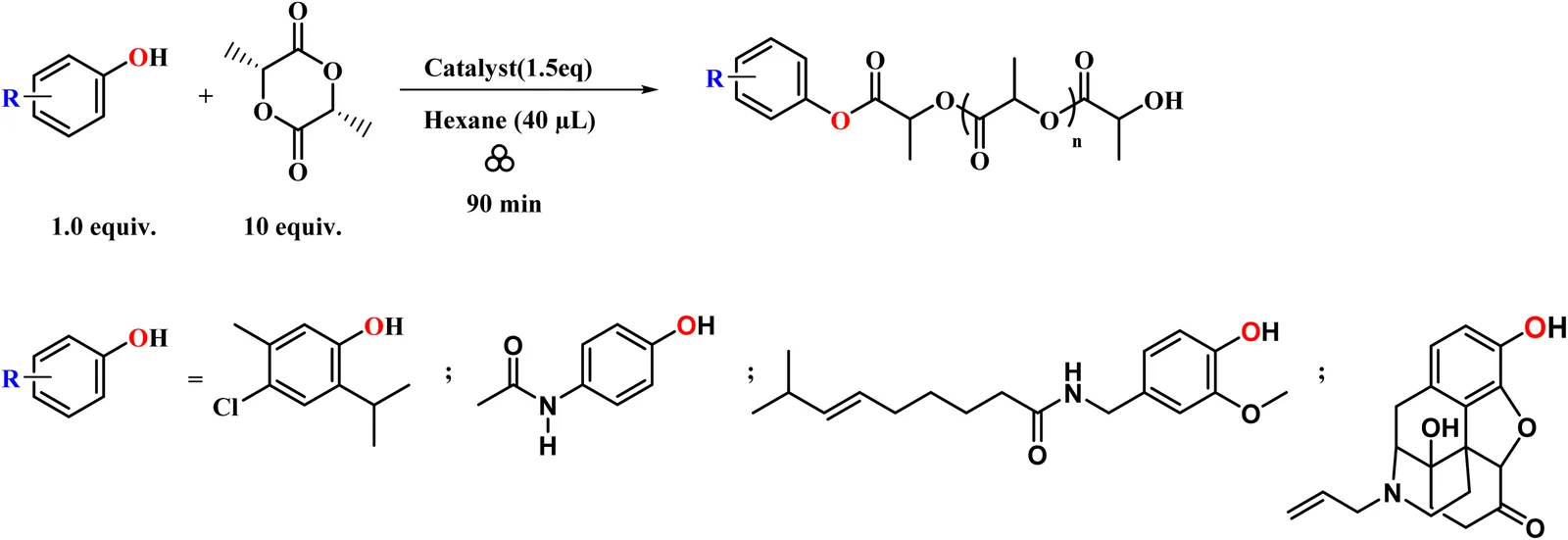
Substrate scope of mechanochemical synthesis of phenolic-drug initiated ROP of l-lactide.

**Table 5:** Substrate scope of different phenolic-drug initiators^a^

Entry	Initiator drug	IE^b^ (%)	Conv.^b^ (%)	DP^c^	*M*_*n*,nmr_ c (kDa)	*M*_*n*,GPC_ d (kDa)	*Đ* d	Drug loadings^e^	Yield^f^ (%)
1	Chlorothymol	50	97	36	2.92	2.68	1.16	6.64	85
2	Acetaminophen	ND	65	61	4.67	3.40	1.26	3.33	52
3	Capsaicin	32	41	33	2.83	2.98	1.20	11.39	39
4	Naloxone	30	48	61	4.86	3.37	1.14	6.94	44

a Polymerization conditions: l-lactide (100 mg, 0.694 mmol, 10 equiv.), phenolic drug (0.0694 mmol, 1 equiv.) in a 5 mL stainless-steel jar with 5 mm (×4) stainless-steel balls at 2500 rpm speed.

b Determined *via* crude ^1^H NMR spectra.

c Determined *via* pure ^1^H NMR analysis (S17–S23).^26^

d Determined *via* GPC.

e eqn (S5).

f Isolated yield. ND: not determined.

Among the phenolic drug initiators, chlorothymol gave the best overall polymerization performance. It afforded 50% IE, 97% lactide conversion, DP ≈ 36, *M*_*n*,GPC_ = 2.68 kDa, *Đ* = 1.16, 6.64% drug loading, and 85% isolated yield (Table 5, entry 1). This performance may result from a good balance between electronic and physical effects. Although the chloro substituent is electron-withdrawing, the methyl and isopropyl groups can donate electron density and increase the hydrophobic character of the initiator. This may improve compatibility with the nonpolar hexane-assisted conditions, while keeping the phenolic O–H group sufficiently accessible for catalyst-assisted initiation.

Acetaminophen gave 65% lactide conversion, DP ≈ 61, *M*_*n*_,_GPC_ = 3.40 kDa, *Đ* = 1.26, 3.33% drug loading, and 52% isolated yield (Table 5, entry 2). The initiation efficiency was not determined for this substrate. The higher DP suggests that acetaminophen can participate in lactide ROP, but initiation was less efficient relative to chain propagation. This may be related to the amide group, which can engage in hydrogen bonding with the catalyst, lactide, or growing polymer chain. Its higher polarity may also reduce compatibility with the nonpolar hexane-assisted reaction medium. Together, these effects may limit effective activation of the phenolic O–H group during milling.

Capsaicin gave lower IE and conversion, 32% and 41%, respectively, but maintained a moderate DP ≈ 33 and showed the highest drug loading, 11.39% (Table 5, entry 3). This high loading is mainly due to the relatively large molecular weight of capsaicin and the moderate PLA chain length. However, the lower IE and conversion suggest that the bulky amide structure and long hydrophobic chain may reduce effective contact between the phenolic O–H group, lactide, and catalyst during milling. Although the methoxy group can donate electron density, the amide group and flexible side chain may introduce competing hydrogen-bonding and mixing effects that limit efficient initiation.

Naloxone showed 30% IE, 48% lactide conversion, DP ≈ 61, *M*_*n*_,_GPC_ = 3.37 kDa, *Đ* = 1.14, 6.94% drug loading, and 44% isolated yield (Table 5, entry 4). The lower of IE and conversion may be attributed to naloxone's rigid, bulky structure, which can hinder access of the phenolic O–H group to the catalyst-activated lactide. This explanation is similar with our previous phenolic-initiated mechanochemical PLA study, which found that naloxone had low initiation efficiency due to steric effects and reduced phenolic group accessibility.^4^ In addition, the tertiary amine and other functional groups may interact with the organocatalyst or affect mixing under the hexane-assisted conditions. Although conversion was moderate, the narrow dispersity indicates that the polymer chains formed in a relatively controlled manner. This result further supports the ability of the BenchMixer/vortexer method to prepare structurally complex phenolic drug–PLA conjugates and agrees with previous reports showing that naloxone can act as an initiator for covalent PLA-based drug delivery systems.^10,18,21^

The GPC results further support the formation of low-molecular-weight drug–PLA conjugates. The *M*_*n*_,_GPC_ values ranged from 2.68 to 3.40 kDa, and the dispersities remained narrow (*Đ* = 1.14–1.26) (Tables 5 and S17–S20). These findings indicate that, although the phenolic drug initiators differ in structure and reactivity, the improved BenchMixer/vortexer system may still provide regulated chain development after initiation. Overall, Table 5 demonstrates how drug structure influences the balance of IE, conversion, DP, drug loading, and yield. Chlorothymol given the best overall performance. Despite a lower conversion rate, capsaicin provided the highest drug loading. In contrast, acetaminophen and naloxone caused greater DP values, most likely due to inefficient start. These data reveal that steric accessibility, electrical effects, polarity, and compatibility with the mechanochemical media all play a key role in phenolic drug-initiated ROP of l-lactide.^27–29^ Future work will focus on improving these parameters, especially drug loading, initiation efficiency, and conversion. Expanding the method to additional phenolic drugs will also help evaluate the broader potential of this BenchMixer/vortexer platform for preparing biodegradable drug–PLA conjugates.

### 2.6. Polymerization of l-lactide initiated by different heteroatom nucleophiles

After evaluating the phenolic and phenolic-drug initiators, three structurally related *para*-methoxy substrates were compared to examine the effect of the initiating heteroatom on BenchMixer/vortexer-assisted ROP of l-lactide. The substrates were 4-methoxyphenol, 4-methoxyaniline, and 4-methoxythiophenol, representing oxygen-, nitrogen-, and sulfur-based initiation, respectively (Table 6). This comparison allowed the role of heteroatom identity to be evaluated under the same optimized mechanochemical conditions.

**Table 6:** Substrate scope of different heteroatoms nucleophiles^a^

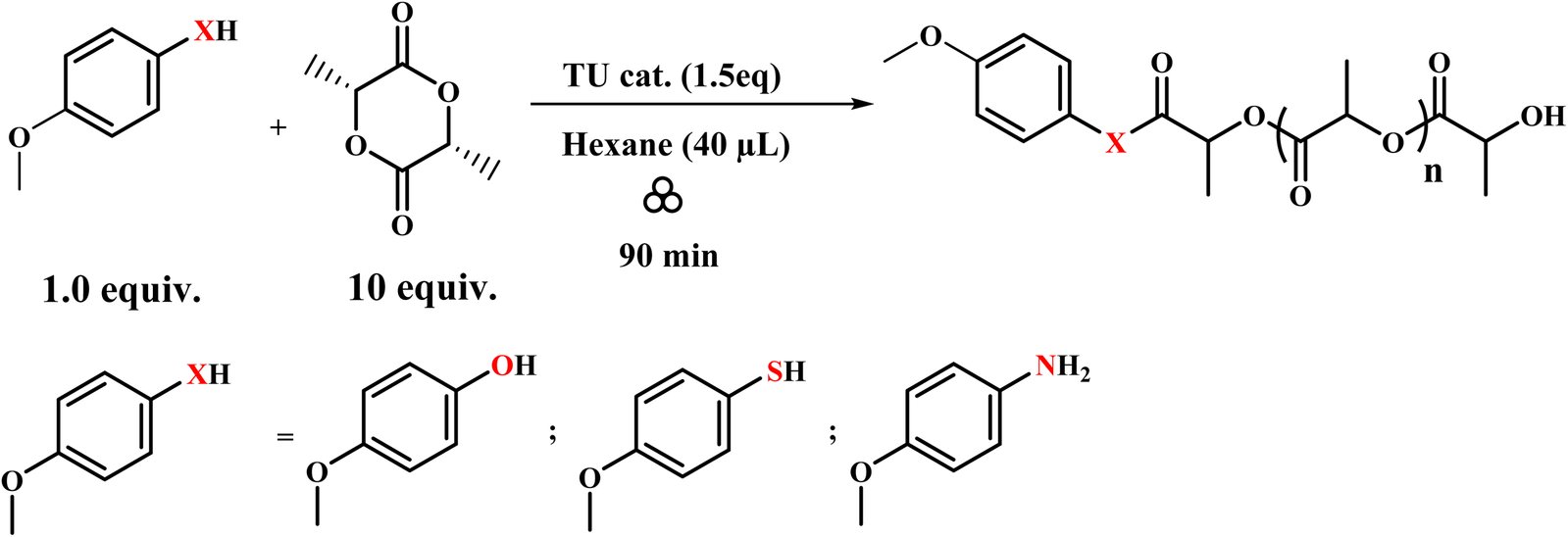								
Entry	Initiator	IE^b^ (%)	Conv.^b^ (%)	DP^c^	*M*_*n*,nmr_ c (kDa)	*M*_*n*,GPC_ d (kDa)	*Đ* d	Yield^e^ (%)
1	*p*-Methoxyphenol	84	>99	25	2.07	2.50	1.18	80
2	*p*-Methoxyaniline	>99	>99	29	2.36	2.25	1.15	82
3	*p*-Methoxythiophenol	60	84	24	2.01	2.14	1.24	58

a Polymerization conditions: l-lactide (100 mg, 0.694 mmol, 10 equiv.), initiator (0.0694 mmol, 1 equiv.) in a 5 mL stainless-steel jar with 5 mm (×4) stainless-steel balls at 2500 rpm speed.

b Determined *via* crude ^1^H NMR spectra.

c Determined *via* pure ^1^H NMR analysis.

d Determined *via* GPC.

e Isolated yield.

Among these substrates, 4-methoxyaniline showed the highest reactivity. It gave >99% IE, >99% conversion, DP ≈ 29, *M*_*n*_,_GPC_ = 2.25 kDa, *Đ* = 1.15, and 82% yield (Table 6, entry 2). This result indicates that the aniline nitrogen is highly effective under the optimized BenchMixer/vortexer conditions. This is likely due to the stronger nucleophilic character of the amine group, which can efficiently initiate lactide ring opening. Similar amine-initiated lactide ring opening, leading to amide end-capped PLA, has also been reported previously.^45^

4-Methoxyphenol also performed well, giving 84% IE, >99% conversion, DP ≈ 25, *M*_*n*,GPC_ = 2.50 kDa, *Đ* = 1.18, and 80% yield (Table 6, entry 1). In contrast, 4-methoxythiophenol showed lower overall performance, with 60% IE, 84% conversion, DP ≈ 24, *M*_*n*,GPC_ = 2.14 kDa, *Đ* = 1.24, and 58% yield (Table 6, entry 3). Although sulfur can be highly polarized and nucleophilic in solutions, the lower performance of 4-methoxythiophenol shows that this reaction is not controlled by nucleophilicity only. However, based on hard–soft acid–base considerations, sulfur is a softer nucleophile, whereas the lactide carbonyl is a relatively hard electrophilic center. This mismatch may make sulfur less favorable for efficient activation and attack at the lactide carbonyl compared with nitrogen- and oxygen-based initiators.^46^

The reactivity trend N > O > S indicates that the reaction is influenced not only by the nucleophilicity of the initiator, but also by how well the initiator interacts with the thiourea catalyst. Under these mechanochemical conditions, the thiourea catalyst can activate lactide and the initiating or propagating chain end through hydrogen bonding. The weaker performance of 4-methoxythiophenol may therefore be related to less efficient activation of the S–H group, weaker hydrogen-bonding interactions, or slower formation of the first thioester-linked PLA species. These factors could reduce initiation efficiency compared with the corresponding aniline and phenol initiators. These factors may reduce initiation efficiency compared with the corresponding aniline and phenol initiators.^28,29,47,48^

Overall, the heteroatom comparison shows that changing only the initiating atom strongly affects lactide initiation, conversion, molecular weight, and yield. The narrow dispersities, *Đ* = 1.15–1.24, indicate that relatively controlled low-molecular-weight polymers formed once initiation occurred. Together with the phenolic-drug substrate scope in Table 5, these results broaden the potential of the BenchMixer/vortexer method beyond simple phenolic initiators. The successful use of drug molecules and different heteroatom nucleophiles suggests that this platform could be extended to new bioactive initiators, including phenolic-, amino-, and thiol-containing drugs. However, the differences in IE, conversion, DP, loading, and yield also show that further optimization will be needed for each drug class. Future studies will focus on improving initiation efficiency, controlling chain length, and increasing drug loading in polymer–drug conjugates.^4,10^

## 3. Experimental section

### 3.1. Materials

(3*S*)-*Cis*-3,6-dimethyl-1,4-dioxane-2,5-dione (l-lactide) was purchased from Sigma-Aldrich. 1-[3,5-Bis(trifluoromethyl)phenyl]-3-[(1*R*,2*R*)-(−)-2-(dimethylamino)cyclohexyl] thiourea catalyst (TU cat.) was obtained from ChemScene. Phenolic initiators were purchased from Fisher Scientific. Naloxone hydrochloride dihydrate was purchased from Sigma-Aldrich and converted to the corresponding free base by acid–base extraction using saturated aqueous NaHCO_3_. All solvents were dried over preactivated alumina and 3 Å molecular sieves. Unless otherwise stated, all reagents were used as received.

### 3.2. Instrumentation

BenchMixer/vortexer experiments were performed using a multi-tube vortexer equipped with SmartSnap stainless-steel grinding jars (5 mL) and four stainless-steel balls (5 mm). ^1^H NMR spectra were recorded on a Bruker Avance 400 MHz spectrometer. Number-average molecular weight (*M*_*n*_), weight-average molecular weight (*M*_w_), and dispersity (*Đ*) were determined by gel permeation chromatography (GPC) using a Tosoh EcoSEC HLC-8320GPC system. Tetrahydrofuran (THF) was used as the mobile phase at a flow rate of 0.35 mL min^−1^. The instrument was calibrated with polystyrene standards, and the data were analyzed using Breeze software.

### 3.3. General representative procedure for the synthesis of phenolic–PLA polymers using the BenchMixer/vortexer (Table 4, entry 1)

In a nitrogen-filled glovebox, l-lactide (100 mg, 0.694 mmol), 4-methoxyphenol (8.59 mg, 0.0694 mmol), thiourea catalyst (42.9 mg, 0.1041 mmol), and hexane (40 µL) were added to a 5 mL stainless-steel grinding jar. Stainless-steel balls (5 mm, 4 balls) were then added, and the jar was sealed, removed from the glovebox, and placed in the BenchMixer/vortexer. The reaction was milled for 90 min at 2500 rpm. Benzoic acid (17 mg, 0.139 mmol) was then added to quench the polymerization, followed by an additional 1 min of milling. The crude product was dissolved in CHCl_3_ (40 mL) and filtered into a 20 mL scintillation vial using a 0.45 µm syringe filter. After solvent removal by rotary evaporation, the product was dissolved in CHCl_3_ (0.5 mL), precipitated into methanol (40 mL), and refrigerated overnight. The solid material was filtered and washed with methanol to remove the starting materials and catalyst residues. The precipitate was dried under a vacuum to afford a white solid (87 mg, 80%). ^1^H NMR (400 MHz, CDCl_3_) *δ* 6.98–7.00 (d, 2H), 6.86–6.89 (d, 2H), 7.07–7.09, 5.30–5.35 (q, 1H), 5.13–5.19 (m, 25H), 4.33–4.38 (q, 1H), 3.79 (s, 3H), 2.69 (s, 1H), 1.66–1.67 (d, 3H), 1.57–1.59 (d, 75H), 1.48–1.50 (d, 3H). GPC: *M*_*n*_ = 2502 Da, *Đ* = 1.18.

## 4. Conclusion

This work addresses the need for a simple, low-cost, and parallel mechanochemical platform for preparing biodegradable polymer–drug conjugates. A multi-tube BenchMixer/vortexer was developed for low-solvent phenolic drug-initiated ROP of l-lactide. The system can process up to 8 sealed reactions simultaneously, enabling rapid optimization and substrate screening under identical conditions.

Using 4-methoxyphenol as a model initiator, the effects of LAG agent, catalyst loading, milling time, and milling frequency were optimized to balance conversion, initiation efficiency, DP, and yield. The optimized hexane-assisted conditions were then applied to substituted phenols, phenolic drugs, and heteroatom analogues. The results showed that initiator structure strongly affects polymerization behavior, with *para*-alkoxy phenols giving the best balance of high conversion, high initiation efficiency, and low DP. The method was successfully extended to chlorothymol, acetaminophen, capsaicin, and naloxone. Chlorothymol showed the best overall performance, giving 97% lactide conversion, DP ≈ 36, *M*_*n*,GPC_ = 2.68 kDa, *Đ* = 1.16, 6.64% drug loading, and 85% isolated yield, while capsaicin gave the highest drug loading of 11.39%. The heteroatom study showed a clear reactivity trend of N > O > S.

Overall, the BenchMixer/vortexer platform offers a practical, low-cost, and solvent-minimized approach to mechanochemical PLA synthesis, supporting rapid screening of bioactive phenolic, amino, and thiol-containing initiators while expanding accessible methods for greener polymer–drug conjugate preparation. This approach may also facilitate the future development of longer-lasting biodegradable drug delivery systems.

## Author contributions

Mohammad A. Ebqa'ai: conceptualization, methodology, investigation, validation, formal analysis, data curation, visualization, supervision, project administration, writing – original draft, writing – review & editing. Kaleb Cossette: methodology. Lydia Park: methodology. Mckalay P. Estep: methodology. K. D. Priyasantha: methodology, validation, resources. Abdullah M. Abu Anzeh: formal analysis, resources, data curation. Weinan Tan: formal analysis, resources, data curation. Sandun B. K. Dias: methodology, formal analysis, resources. Toby L. Nelson: validation, formal analysis, data curation, resources, writing – review & editing.

## Conflicts of interest

The authors declare no conflict of interest.

## Supplementary Material

RA-OLF-D6RA07580K-s001

## Data Availability

The data supporting the findings of this study are available within the article and its supplementary information (SI). Supplementary information is available. See DOI: https://doi.org/10.1039/d6ra07580k.
