# Supplementary material for: Parallel mechanochemical synthesis of phenolic drug–poly(lactic acid) conjugates using a multi-tube BenchMixer/vortexer
Source: RSC Adv. 2026 Oct 26. Online ahead of print. doi: 10.1039/d6ra07580k (PMC13702836; doi:10.1039/d6ra07580k)
Supplement: Supplementary file 1 [file RA-OLF-D6RA07580K-s001.pdf]

Supplementary Information (SI) for RSC Advances.
This journal is © The Royal Society of Chemistry

"Supporting Information"

Parallel Mechanochemical Synthesis of Phenolic Drug–Poly(lactic acid) Conjugates Using a Multi-Tube BenchMixer/Vortexer

Mohammad A. Ebqa'ai*[a], Kaleb Cossette[b], Lydia Park[b], Mckalay P. Estep[b], KD Priyasantha[b], Abdullah M. Abu Anzeh[c], Weinan Tan[d], Sandun B.K Dias[e,f], Toby L. Nelson[f]

[a] Department of Chemistry, Yarmouk University, P.O. BOX 566, Irbid 21163, Jordan (mebqaai@yu.edu.jo)
[b] Department of Chemistry, Physics, and Engineering, Cameron University, Lawton, Ok 73505, USA.
[c] Faculty of Science, Coker University, Hartsville, SC 29550, USA.
[d] Department of Chemistry, University of North Texas, Denton, TX 76201, USA.
[e] Bredesen Center for Interdisciplinary Research and Graduate Education, The University of Tennessee, Knoxville, TN 37996, USA
[f] Oak Ridge Innovation Institute, The University of Tennessee, Knoxville, TN 37996, USA

Calculations and Equations:

Degree of polymerization determination (DP)

DP = number of repeating units (n) that estimated from ratio of integrals of *ortho*-proton (**h**) from the phenolic end group to the lactyl methine proton (**a**) from lactic acid polymer chain. (**eq S1**)

ROP conversion determination (Conv. %)

Conversions were calculated by the ratio of integrals obtained for the methyl protons (CH_3) of poly (lactic acid) and unreacted L-lactide from the crude ^{1}H NMR spectra according to the following equation:

$$\%\ \text{conversion} = \frac{\text{Integral of polymer methyl } (CH_3) \text{ protons}}{\left[\text{Integral of polymer methyl } (CH_3) \text{ protons} + \text{Integral of unreacted monomer methyl } (CH_3) \text{ protons}\right]} \times 100 \qquad \text{(eq S2)}$$

Initiator efficiency determination (IE %)

IE values were calculated by the ratio of integrals obtained for the ortho-CH protons of phenols bound-polymer and unreacted phenols from the crude ^{1}H NMR spectra according to the following equation:

$$\%\ \text{IE} = \frac{\text{Integral of phenols-bound polymer ortho- (CH) resonance}}{\left[\text{Integral of phenols-bound polymer ortho- (CH) resonance} + \text{Integral of unreacted phenols ortho- (CH) resonance}\right]} \times 100 \qquad \text{(eq S3)}$$

$M_{n,NMR}$ determination:

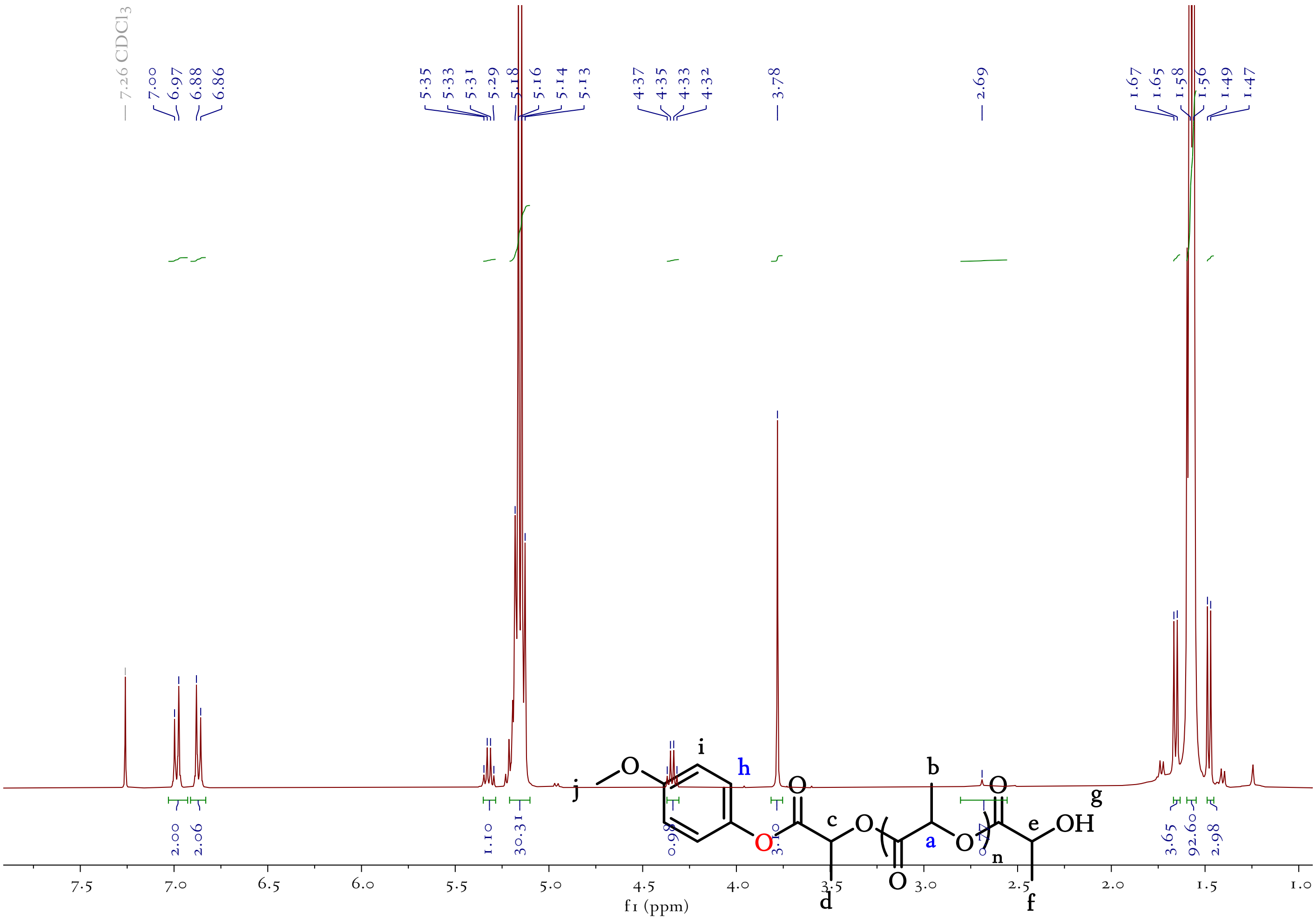

$M_{n,\ NMR}$ = M.W$_{initiator}$ + M.W $_{beginning\ group}$ (72) + (M.W $_{repeating\ unit}$ (72) x DP) + (M.W $_{ending\ group}$ (72))

(**eq S4**).[1]

Drug loading determination:

For an estimation for drug loading, a standard ^{1}H NMR-based molecular weight analysis technique was used. Drug loading can be determined based on equation below:[2]

% drug loading = M.W drug / [(DP x72) + M.W drug] x 100% (**eq S5**)

Figure S1: ^{1}H NMR of 4-methoxyphenol-PLA polymer, **Table 1, entry 4**

a

b

^{1}H NMR chemical shift (δ) and coupling constant (J) values:

^{1}H NMR (400 MHz, $CDCl_3$) δ 6.99 (d, J = 9.0 Hz, 2H), 6.87 (d, J = 9.1 Hz, 2H), 5.32 (q, J = 7.1 Hz, 1H), 5.15 (q, J = 7.1 Hz, 30H), 4.34 (q, J = 6.9 Hz, 1H), 3.78 (s, 3H), 2.69 (s, 1H), 1.66 (d, J = 7.1 Hz, 3H), 1.57 (d, J = 7.1 Hz, 93H), 1.48 (d, J = 6.8 Hz, 3H).

Table S1. Conducting three reactions simultaneously for the same reaction conditions[a]

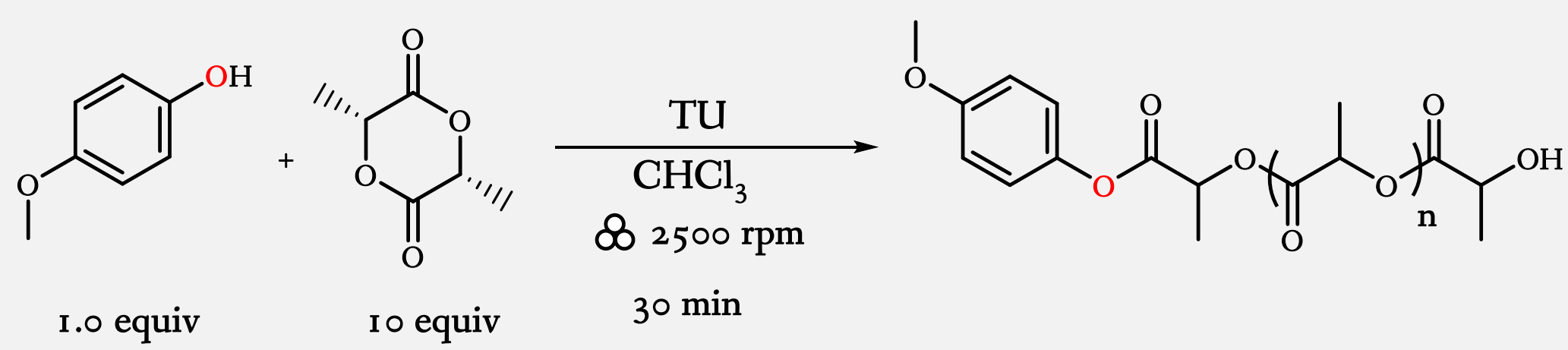

Entry	TU (equiv.)	$CHCl_3$ (μL)	IE[b] (%)	Conv.[b] (%)	DP[b]	Yield[c] (%)
1	0.5	40	65	71	30	42
2	0.5	40	66	70	30	38
3	0.5	40	64	72	29	41

[a] Polymerization conditions: ʟ-Lactide (100 mg, 0.694 mmol, 10 equiv.), 4-methoxy Phenol (8.60 mg, 0.0694 mmol, 1 equiv.) in a 5 mL stainless-steel jar with 5 mm (x4) stainless-steel balls for 30 min at 2500 rpm speed. [b] Determined via ^{1}H NMR. [c] Isolated yield.

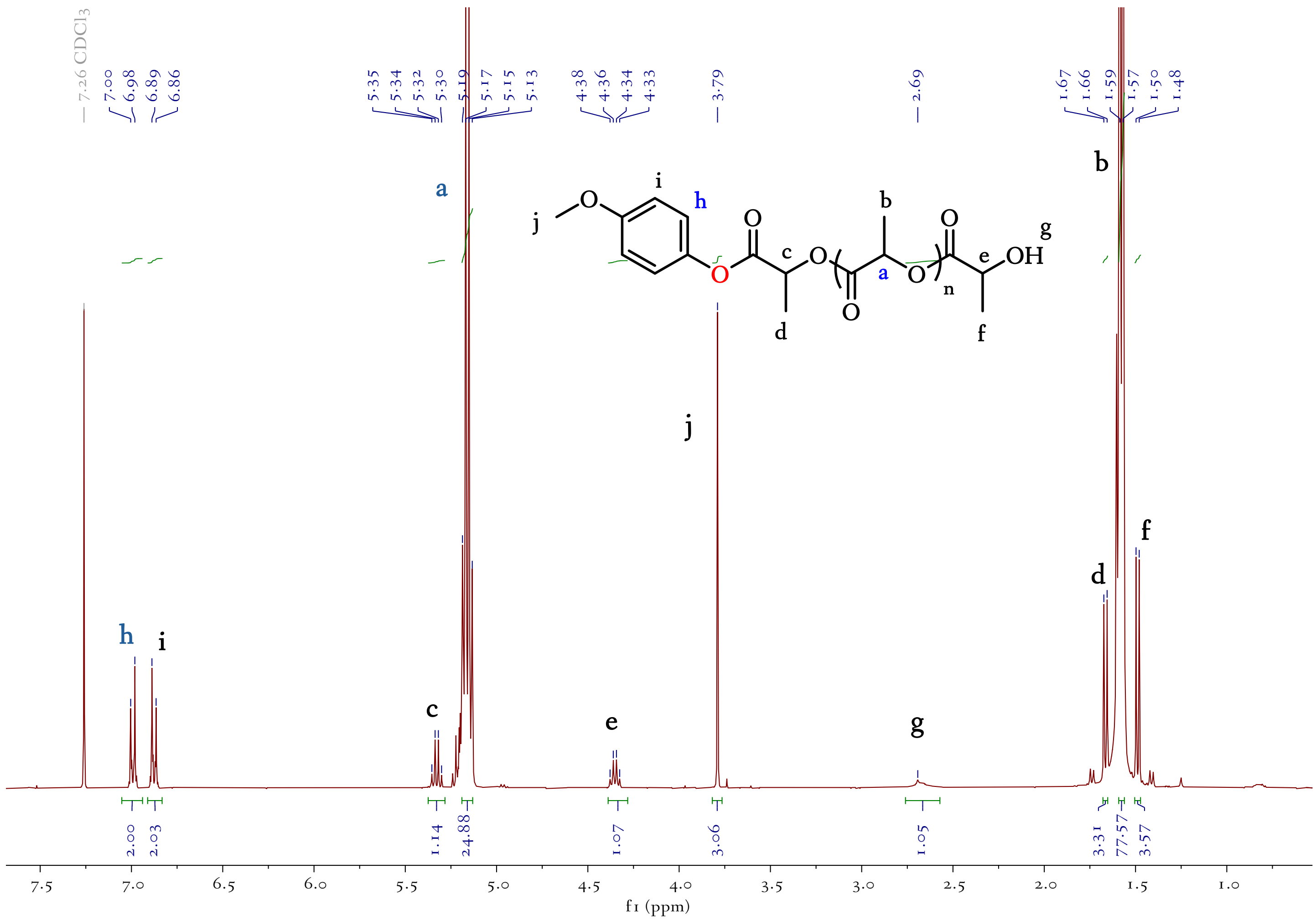

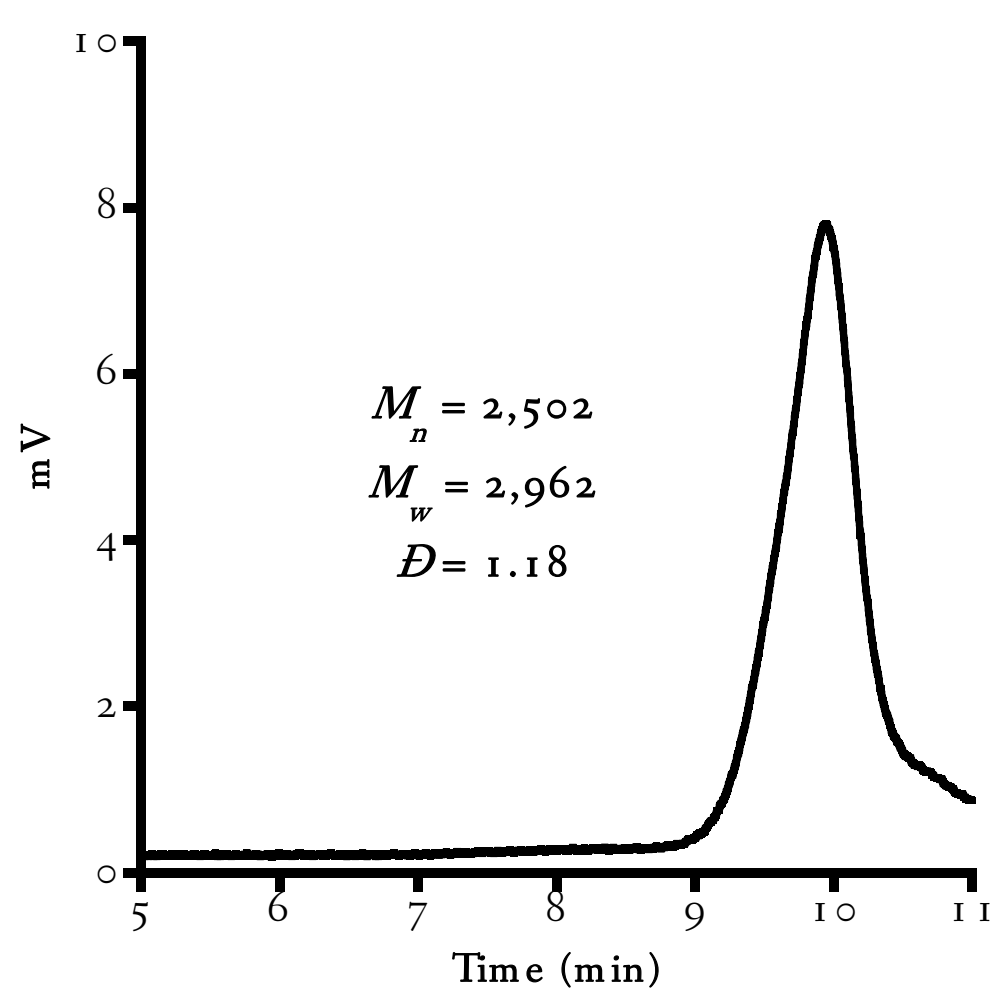

Figure S2: Table 4, entry 1, *p*-methoxyphenol-PLA polymer, ^{1}H NMR (above) and GPC (below).

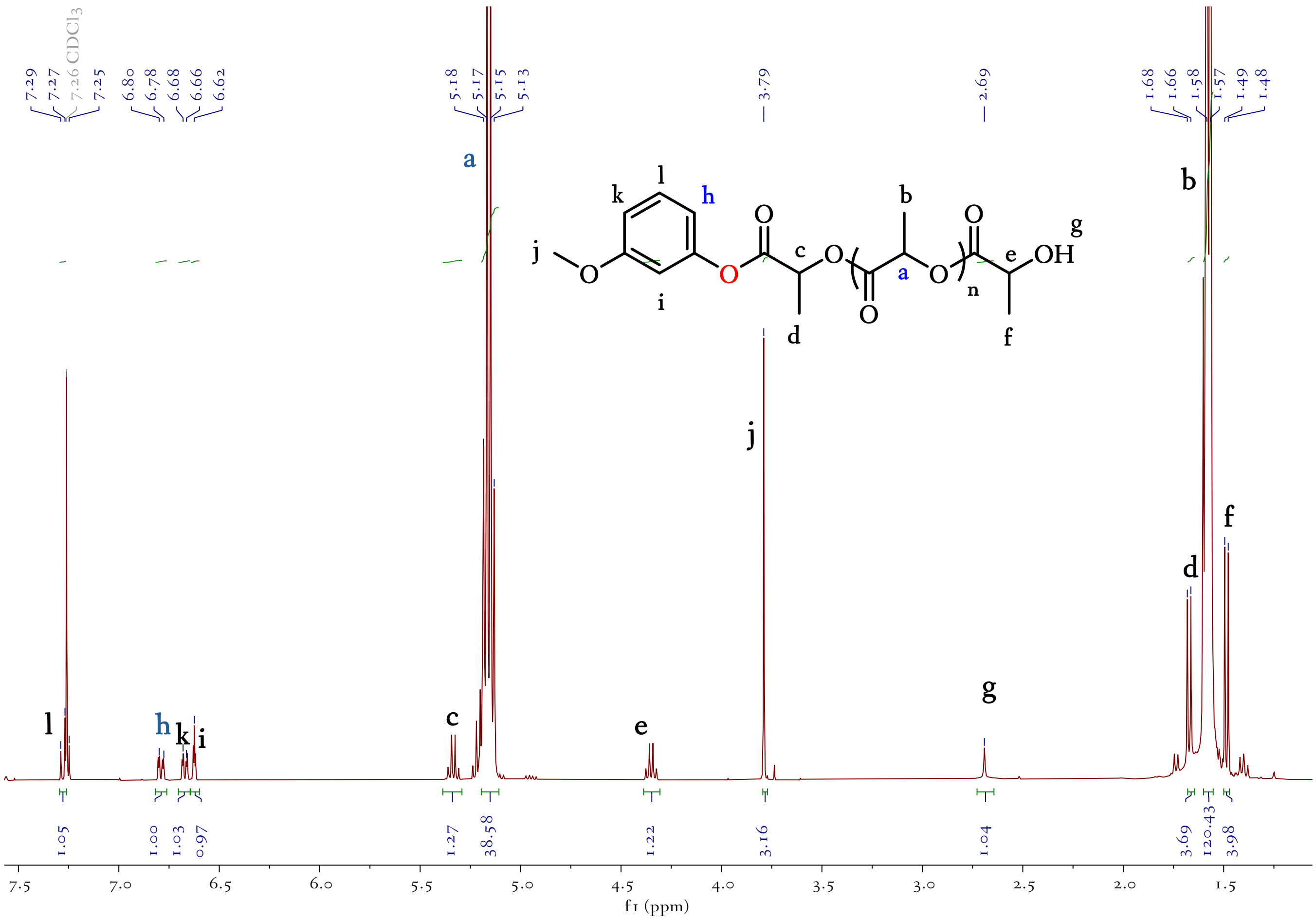

Figure S3: ^{1}H NMR of *m*-methoxyphenol-PLA polymer, **Table 4, entry 2**.

^{1}H NMR chemical shift (δ) and coupling constant (J) values:

^{1}H NMR (400 MHz, $CDCl_3$) δ 7.27 (t, *J* = 8.2 Hz, 1H), 6.79 (d, *J* = 9.4 Hz, 1H), 6.67 (d, *J* = 6.8 Hz, 1H), 6.62 (s, 1H), 5.33 (q, *J* = 7.1 Hz, 1H), 5.16 (q, *J* = 7.1 Hz, 39H), 4.35 (q, *J* = 6.9 Hz, 1H), 3.79 (s, 3H), 2.69 (s, 1H), 1.67 (d, *J* = 7.1 Hz, 3H), 1.57 (d, *J* = 7.1 Hz, 120H), 1.49 (d, *J* = 6.9 Hz, 3H).

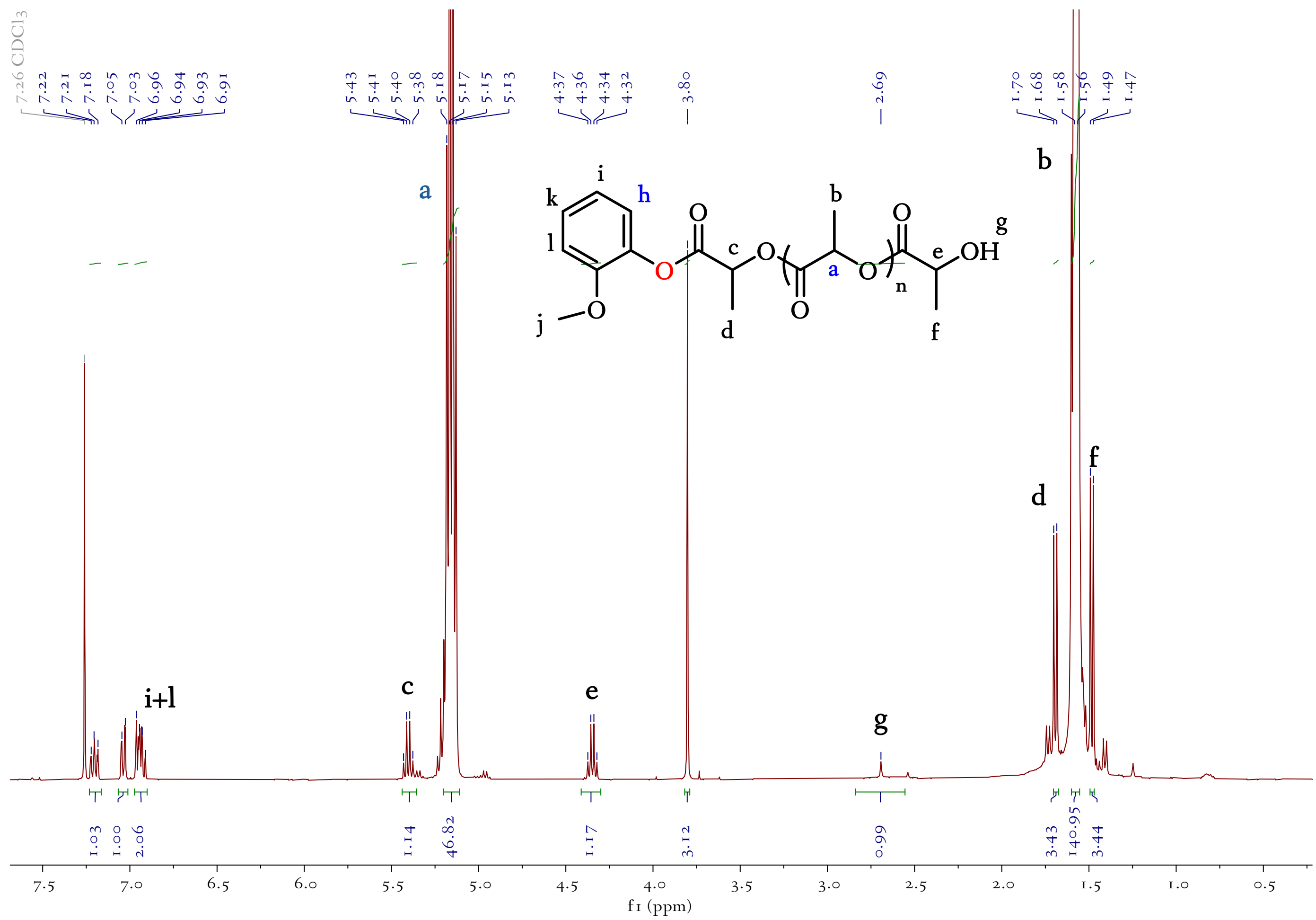

Figure S4: ^{1}H NMR of *o*-methoxyphenol-PLA polymer, **Table 4, entry 3**.

^{1}H NMR chemical shift (δ) and coupling constant (J) values:

^{1}H NMR (400 MHz, $CDCl_3$) δ 7.20 (t, *J* = 7.8 Hz, 1H), 7.04 (d, *J* = 7.8 Hz, 1H), 6.95 (t, *J* = 6.9 Hz, 1H), 6.92 (d, *J* = 7.6 Hz, 1H), 5.40 (q, *J* = 7.1 Hz, 2H), 5.16 (q, *J* = 7.1 Hz, 47H), 4.35 (q, *J* = 6.9 Hz, 1H), 3.80 (s, 6H), 2.69 (s, 1H), 1.69 (d, *J* = 7.1 Hz, 3H), 1.57 (d, *J* = 7.1 Hz, 141H), 1.48 (d, *J* = 6.9 Hz, 3H).

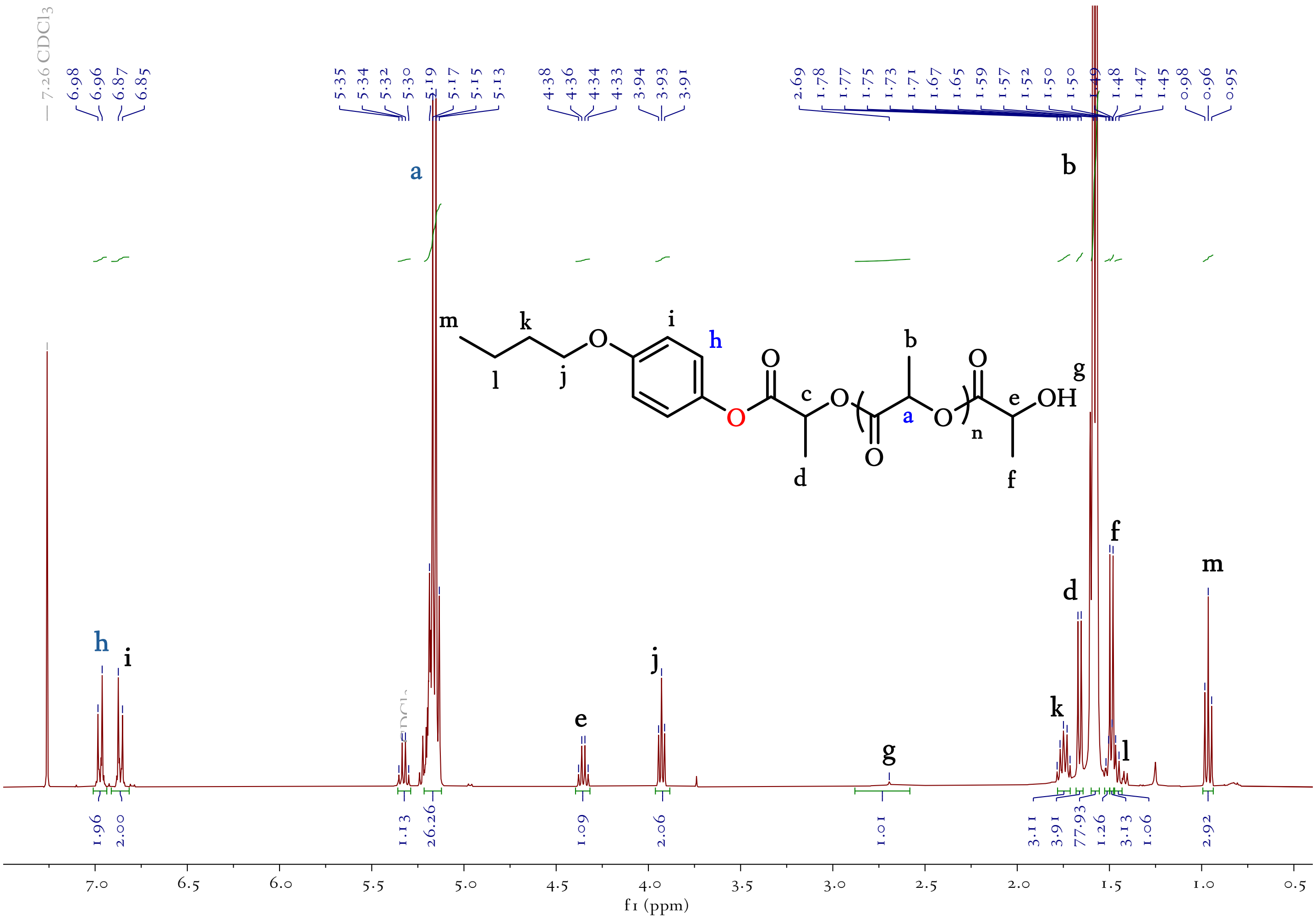

Figure S5: ^{1}H NMR of *p*-Butoxyphenol-PLA polymer, **Table 4, entry 4**.

^{1}H NMR chemical shift (δ) and coupling constant (J) values:

^{1}H NMR (400 MHz, $CDCl_3$) δ 6.97 (d, *J* = 9.1 Hz, 1H), 6.86 (d, *J* = 9.1 Hz, 1H), 5.33 (q, *J* = 7.1 Hz, 1H), 5.16 (q, *J* = 7.1 Hz, 26H), 4.35 (q, *J* = 6.9 Hz, 1H), 3.93 (t, *J* = 6.5 Hz, 1H), 2.69 (s, 1H), 1.85 – 1.69 (m, 2H), 1.66 (d, *J* = 7.0 Hz, 2H), 1.58 (d, *J* = 7.1 Hz, 78H), 1.49 (d, *J* = 6.9 Hz, 3H), 1.46 (m, 2H), 0.96 (t, *J* = 7.4 Hz, 3H).

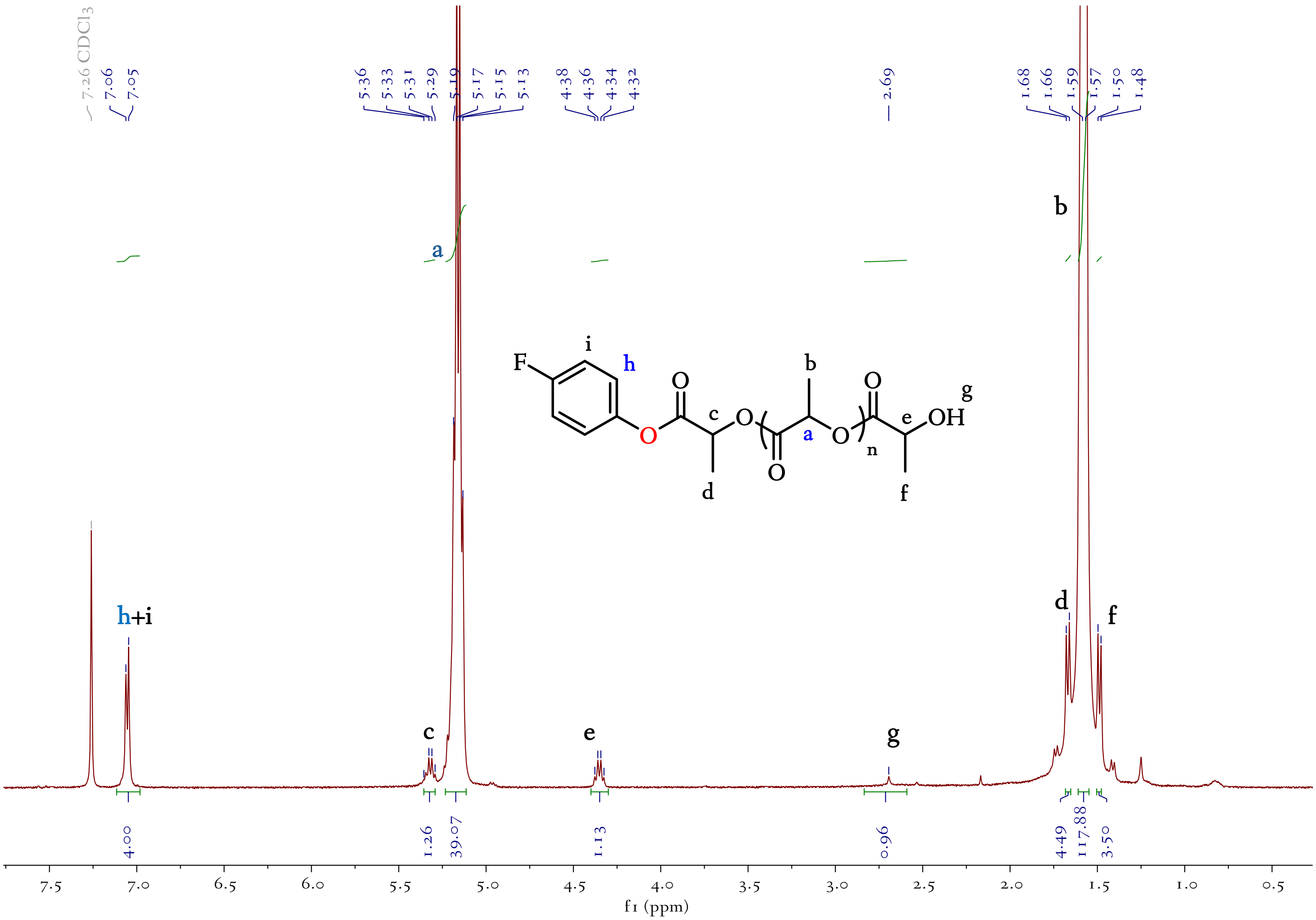

Figure S6: ^{1}H NMR of *p*-Fluorophenol-PLA polymer, **Table 4, entry 5**.

^{1}H NMR chemical shift (δ) and coupling constant (J) values:

^{1}H NMR (400 MHz, $CDCl_3$) δ 7.05 (d, *J* = 6.3 Hz, 4H), 5.31 (q, *J* = 7.2 Hz, 1H), 5.16 (d, *J* = 7.1 Hz, 39H), 4.35 (q, *J* = 6.9 Hz, 1H), 2.69 (s, 1H), 1.67 (d, *J* = 7.1 Hz, 3H), 1.58 (d, *J* = 7.0 Hz, 117H), 1.49 (d, *J* = 6.9 Hz, 3H).

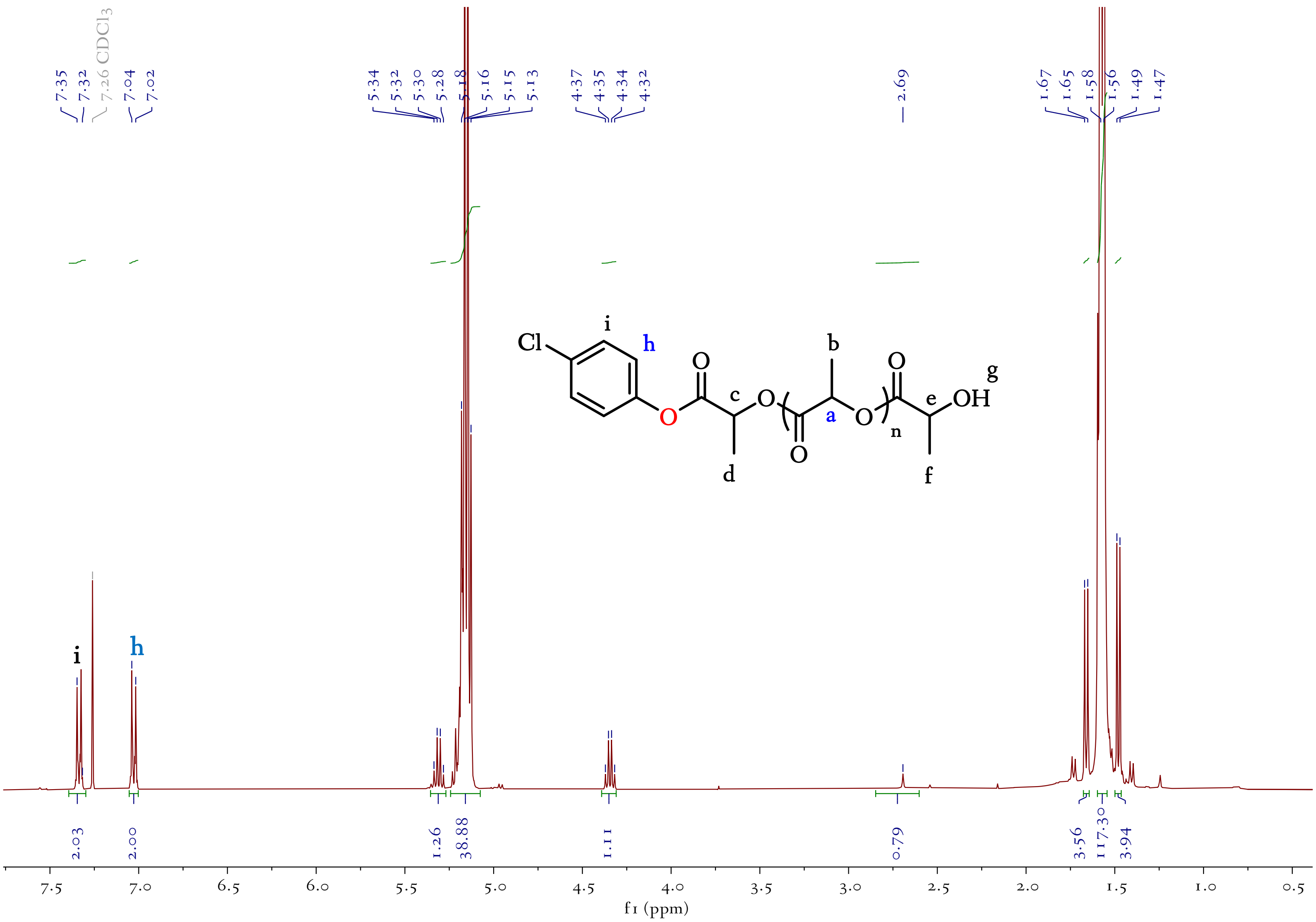

Figure S7: ^{1}H NMR of *p*-Chlorophenol-PLA polymer, **Table 4, entry 6**.

^{1}H NMR chemical shift (δ) and coupling constant (J) values:

^{1}H NMR (400 MHz, $CDCl_3$) δ 7.34 (d, *J* = 8.9 Hz, 2H),7.03 (d, *J* = 8.9 Hz, 2H), 5.31 (q, *J* = 7.1 Hz, 1H), 5.15 (q, *J* = 7.1 Hz, 39H), 4.34 (q, *J* = 6.9 Hz, 1H), 2.69 (s, 1H), 1.66 (d, *J* = 7.1 Hz, 3H), 1.57 (d, *J* = 7.0 Hz, 117H), 1.48 (d, *J* = 6.9 Hz, 3H).

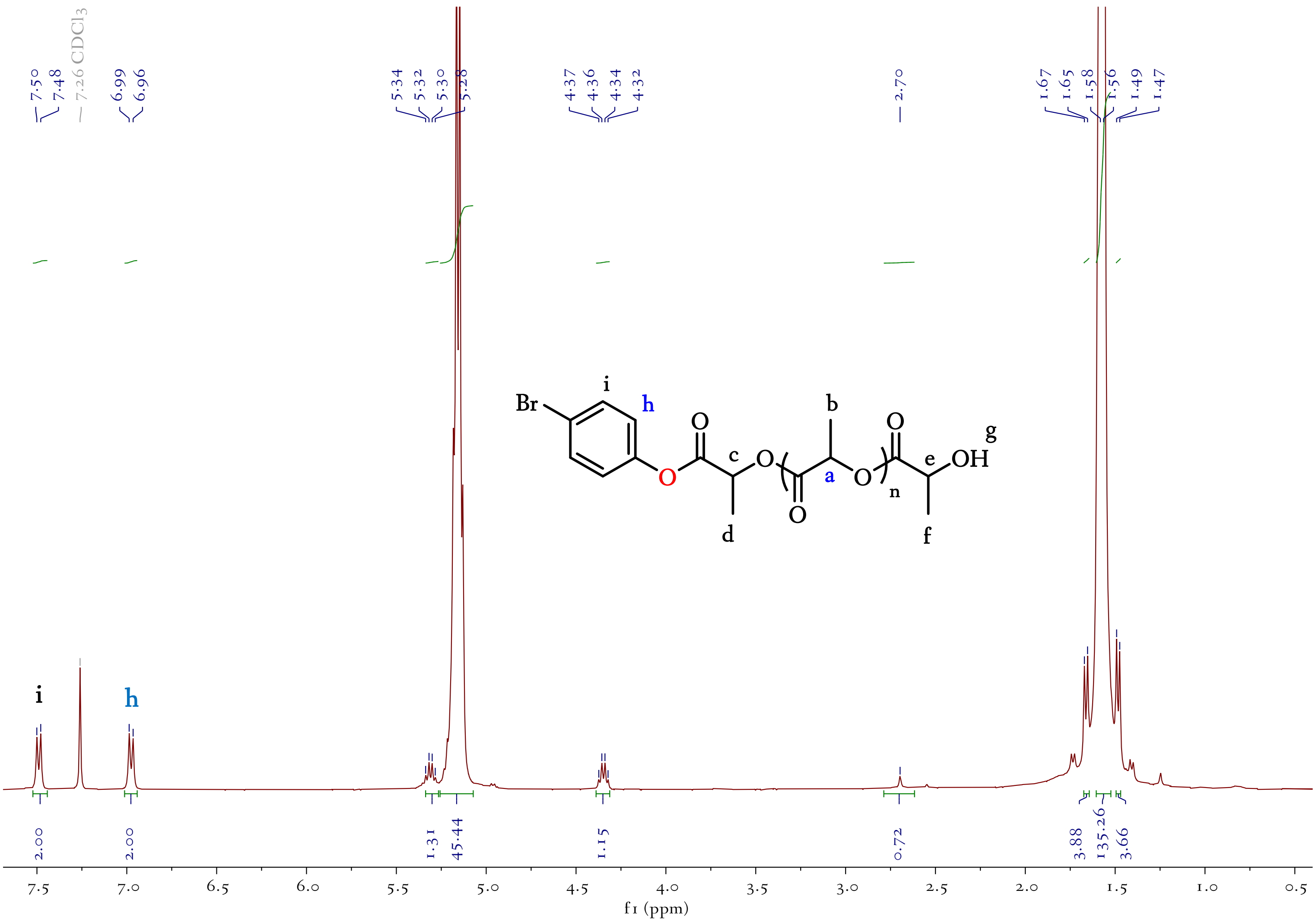

Figure S8: ^{1}H NMR of *p*-Bromophenol-PLA polymer, **Table 4, entry 7**.

^{1}H NMR chemical shift (δ) and coupling constant (J) values:

^{1}H NMR (400 MHz, $CDCl_3$) δ 7.49 (d, *J* = 8.7 Hz, 2H), 6.98 (d, *J* = 8.8 Hz, 2H), 5.31 (q, *J* = 7.2 Hz, 1H), 5.16 (d, *J* = 7.1 Hz, 45H), 4.35 (q, *J* = 6.8 Hz, 1H), 2.70 (s, 1H), 1.66 (d, *J* = 7.0 Hz, 3H), 1.57 (d, *J* = 7.1 Hz, 135H), 1.48 (d, *J* = 6.9 Hz, 3H).

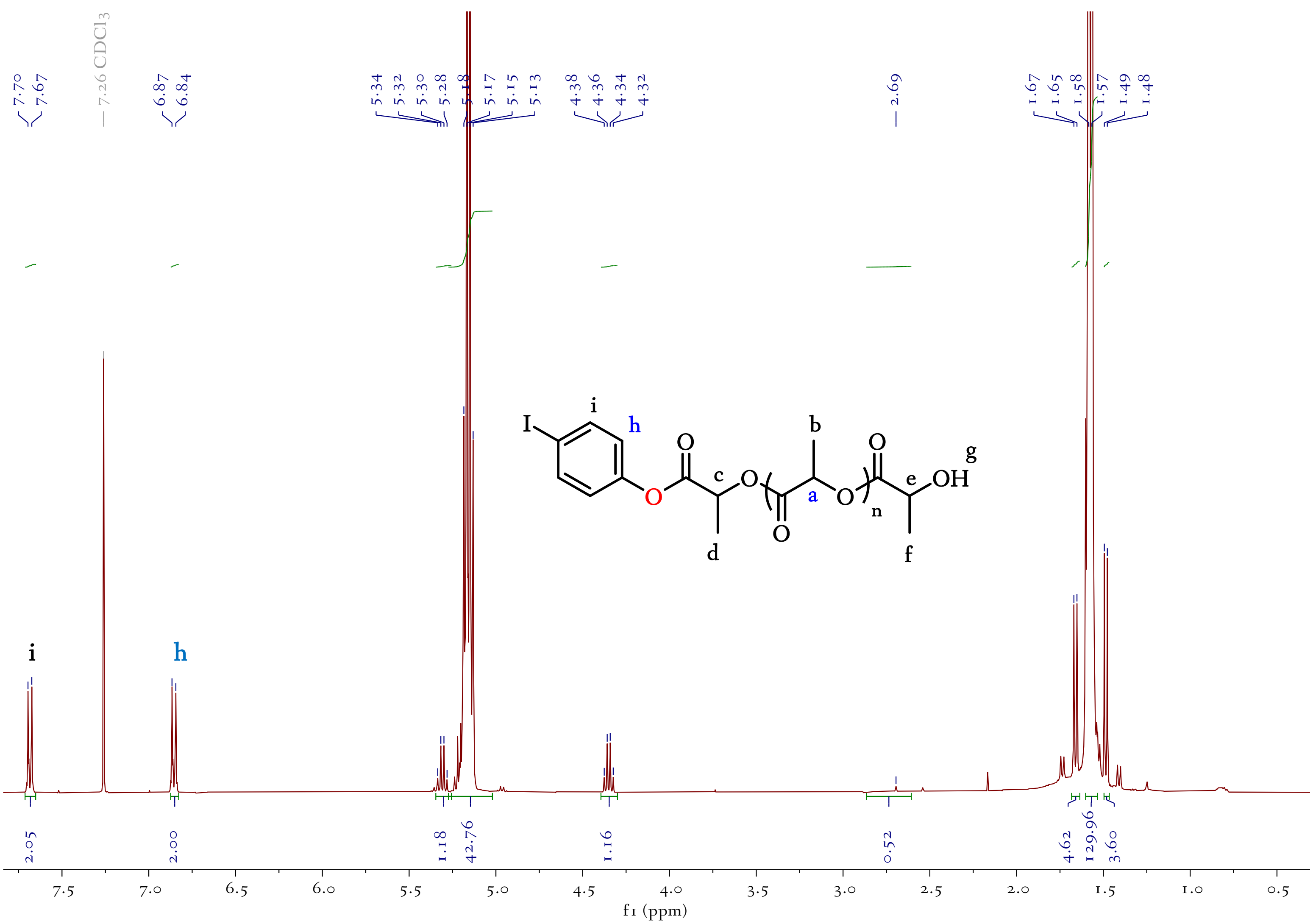

Figure S9: ^{1}H NMR of *p*-Iodophenol-PLA polymer, **Table 4, entry 8**.

^{1}H NMR chemical shift (δ) and coupling constant (J) values:

^{1}H NMR (400 MHz, $CDCl_3$) δ 7.68 (d, *J* = 8.8 Hz, 2H), 6.86 (d, *J* = 8.9 Hz, 2H), 5.31 (q, *J* = 7.1 Hz, 1H), 5.16 (q, *J* = 7.1 Hz, 43H), 4.35 (q, *J* = 6.9 Hz, 1H), 2.69 (s, 1H), 1.66 (d, *J* = 7.0 Hz, 3H), 1.58 (d, *J* = 7.1 Hz, 129H), 1.49 (d, *J* = 6.9 Hz, 3H).

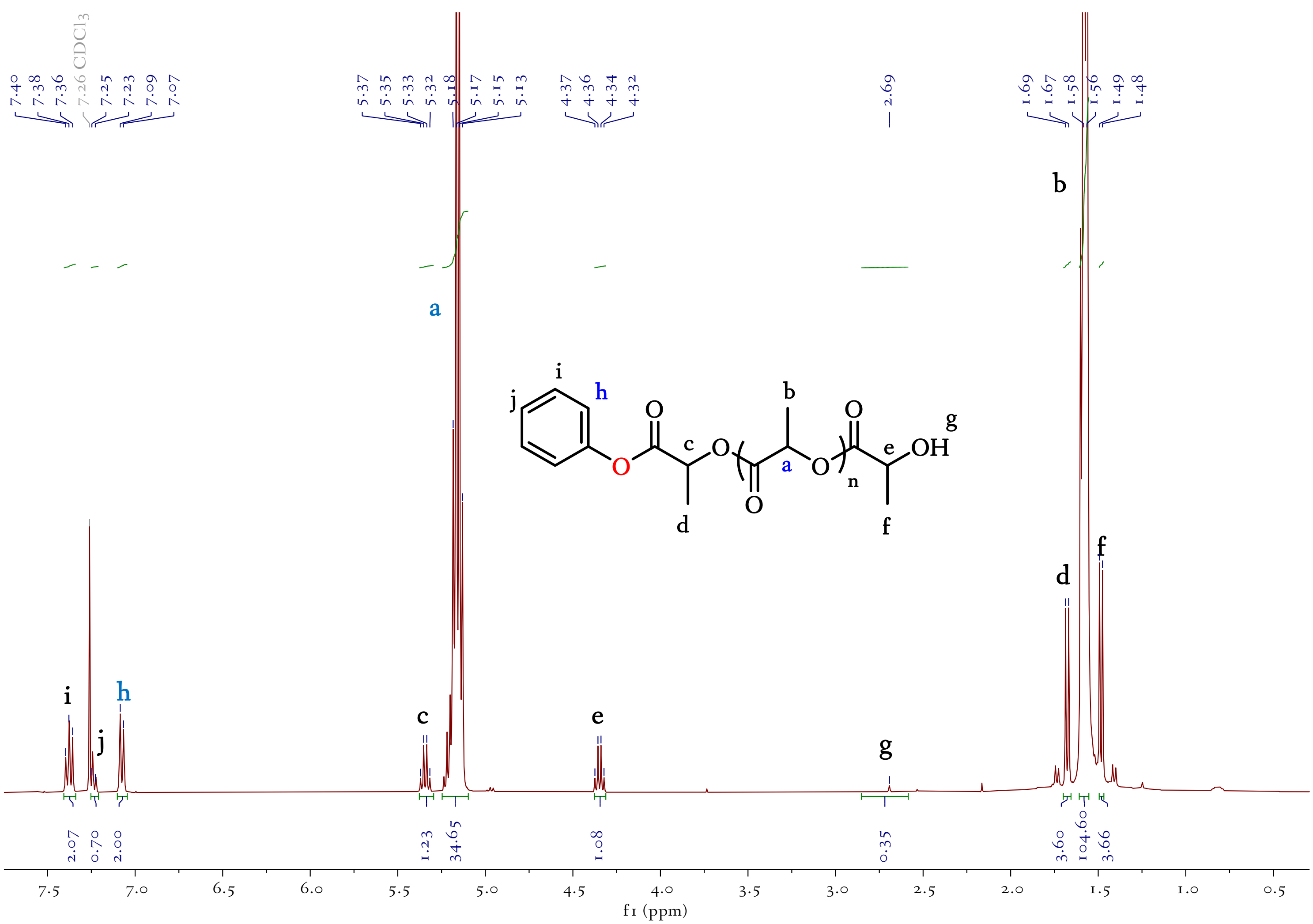

Figure S10: ^{1}H NMR of phenol-PLA polymer, **Table 4, entry 9**.

^{1}H NMR chemical shift (δ) and coupling constant (J) values:

^{1}H NMR (400 MHz, $CDCl_3$) δ 7.38 (t, *J* = 7.9 Hz, 2H), δ 7.24 (t, *J* = 7.4 Hz, 1H), 7.23 (d, *J* = 7.4 Hz, 2H), 5.34 (q, *J* = 7.1 Hz, 1H), 5.16 (q, *J* = 7.1 Hz, 35H), 4.35 (q, *J* = 6.9 Hz, 1H), 2.69 (s, 1H), 1.68 (d, *J* = 7.1 Hz, 3H), 1.57 (d, *J* = 7.1 Hz, 105H), 1.48 (d, *J* = 6.9 Hz, 3H).

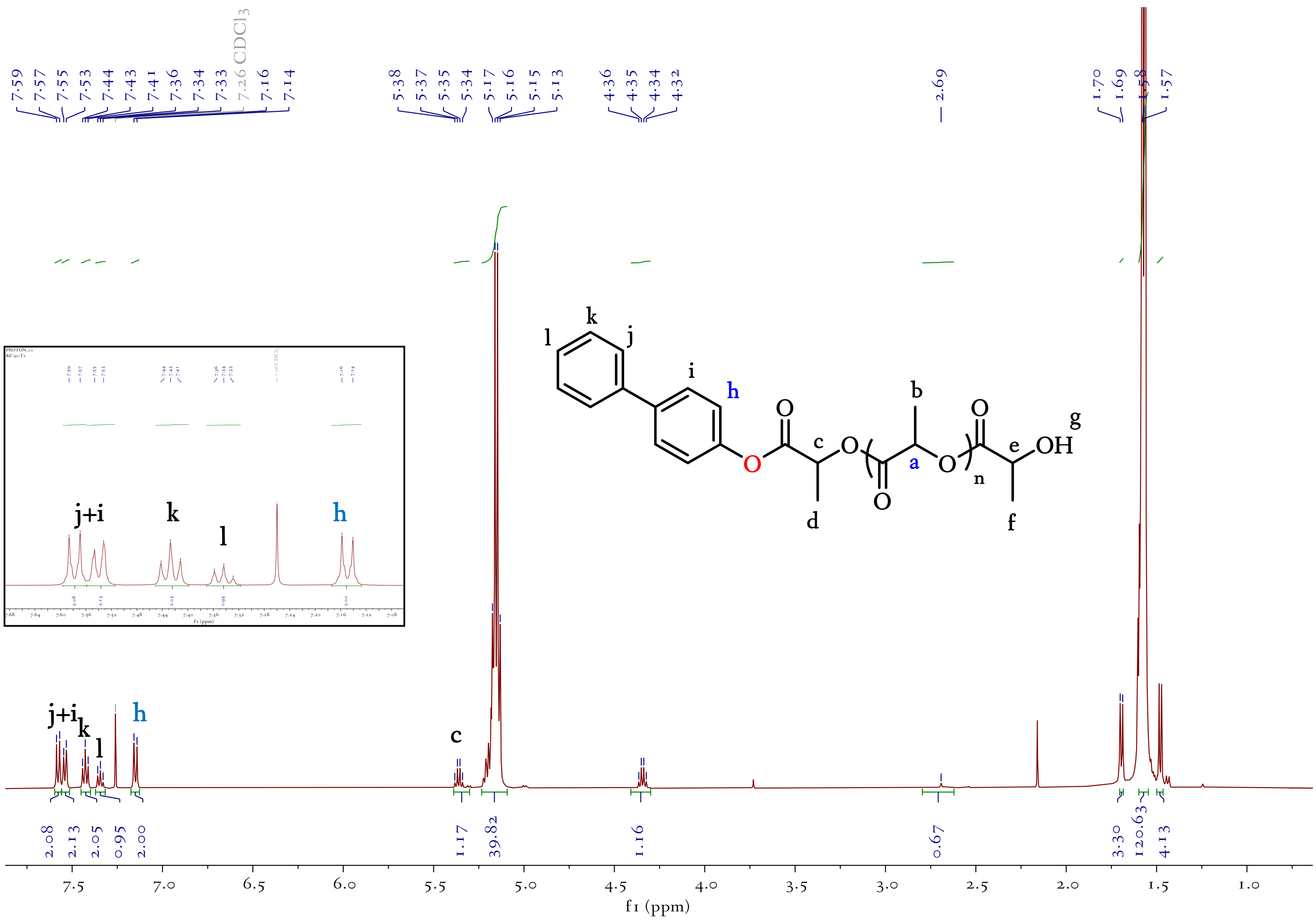

Figure S11: ^{1}H NMR of *p*-phenylphenol-PLA polymer, **Table 4, entry 10**.

^{1}H NMR chemical shift (δ) and coupling constant (J) values:

^{1}H NMR (400 MHz, $CDCl_3$) δ 7.58 (d, *J* = 8.7 Hz, 2H), 7.54 (d, *J* = 7.0 Hz, 2H), 7.43 (t, *J* = 7.6 Hz, 2H), 7.34 (t, *J* = 7.4 Hz, 1H), 7.15 (d, *J* = 8.6 Hz, 2H), 5.36 (q, *J* = 7.2 Hz, 1H), 5.15 (q, *J* = 7.1 Hz, 40H), 4.34 (q, *J* = 6.9 Hz, 1H), 2.69 (s, 1H), 1.69 (d, *J* = 7.1 Hz, 1H), 1.57 (d, *J* = 4.3 Hz, 120H), 1.48 (d, *J* = 6.9 Hz, 3H).

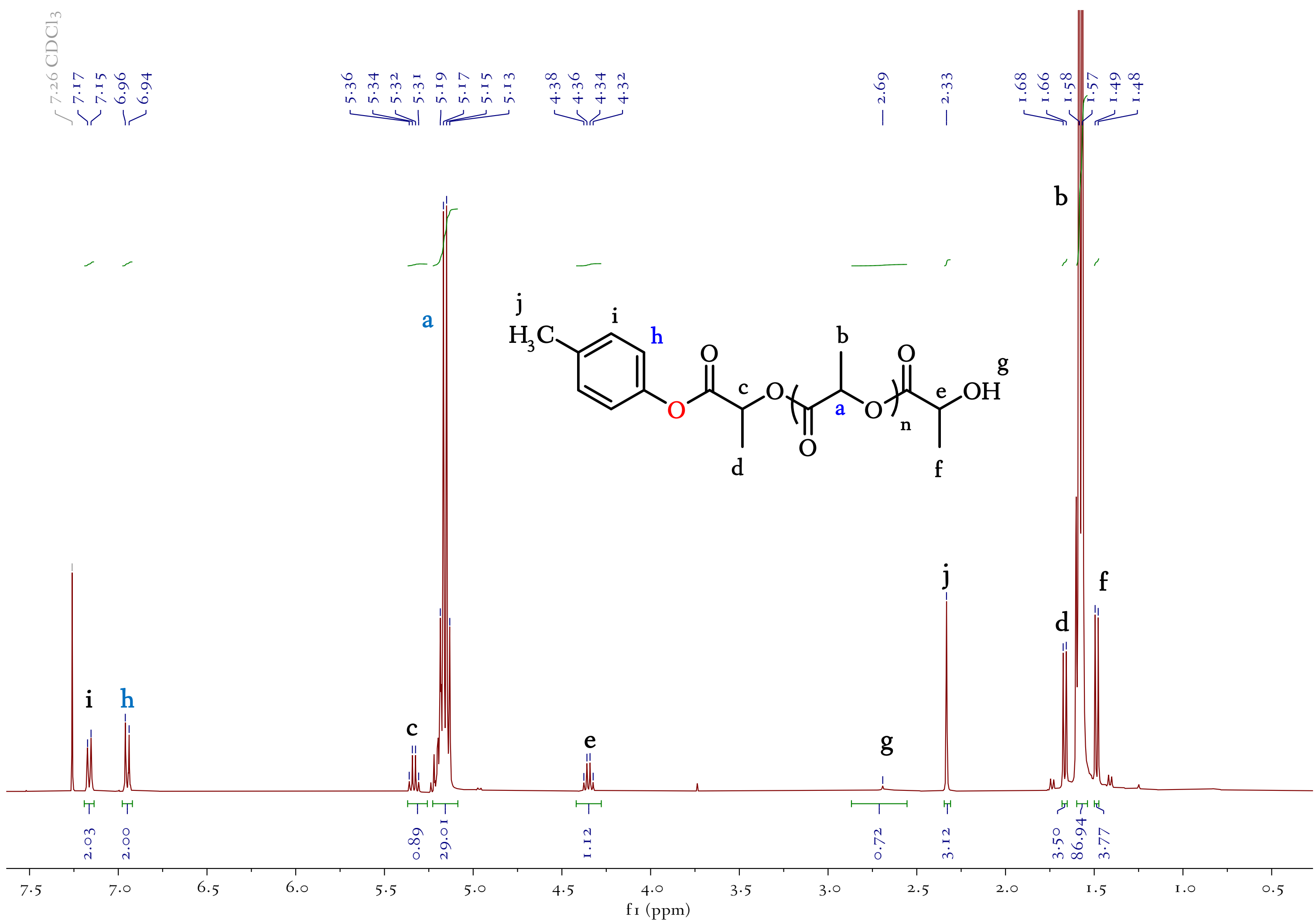

Figure S12: ^{1}H NMR of *p*-Methylphenol-PLA polymer, **Table 4, entry 11**.

^{1}H NMR chemical shift (δ) and coupling constant (J) values:

^{1}H NMR (400 MHz, $CDCl_3$) δ 7.16 (d, *J* = 7.8 Hz, 2H), 6.95 (d, *J* = 8.6 Hz, 2H), 5.33 (q, *J* = 7.1 Hz, 1H), 5.16 (q, *J* = 7.1 Hz, 29H), 4.35 (q, *J* = 7.0 Hz, 1H), 2.69 (s, 1H), 2.33 (s, 3H), 1.67 (d, *J* = 7.0 Hz, 3H), 1.58 (d, *J* = 7.0 Hz, 87H), 1.49 (d, *J* = 6.9 Hz, 3H).

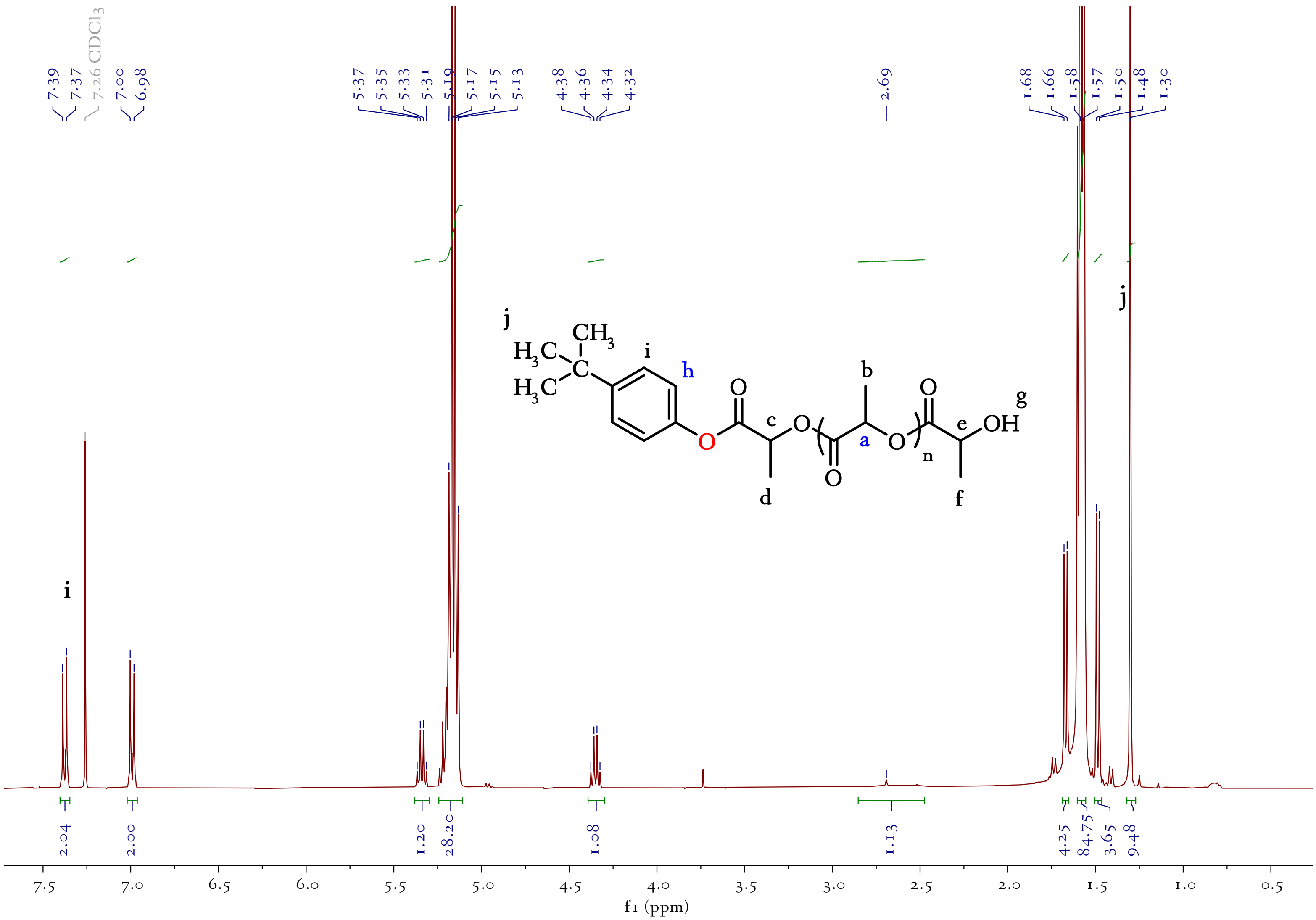

Figure S13: ^{1}H NMR of *p*-tert-butylphenol-PLA polymer, **Table 4, entry 12**.

^{1}H NMR chemical shift (δ) and coupling constant (J) values:

^{1}H NMR (400 MHz, $CDCl_3$) δ 7.38 (d, *J* = 8.8 Hz, 2H), 6.99 (d, *J* = 8.7 Hz, 2H), 5.34 (q, *J* = 7.1 Hz, 1H), 5.16 (q, *J* = 7.1 Hz, 28H), 4.35 (q, *J* = 6.9 Hz, 1H), 2.69 (s, 1H), 1.67 (d, *J* = 7.1 Hz, 3H), 1.58 (d, *J* = 7.1 Hz, 84H), 1.49 (d, *J* = 6.9 Hz, 3H), 1.30 (s, 9H).

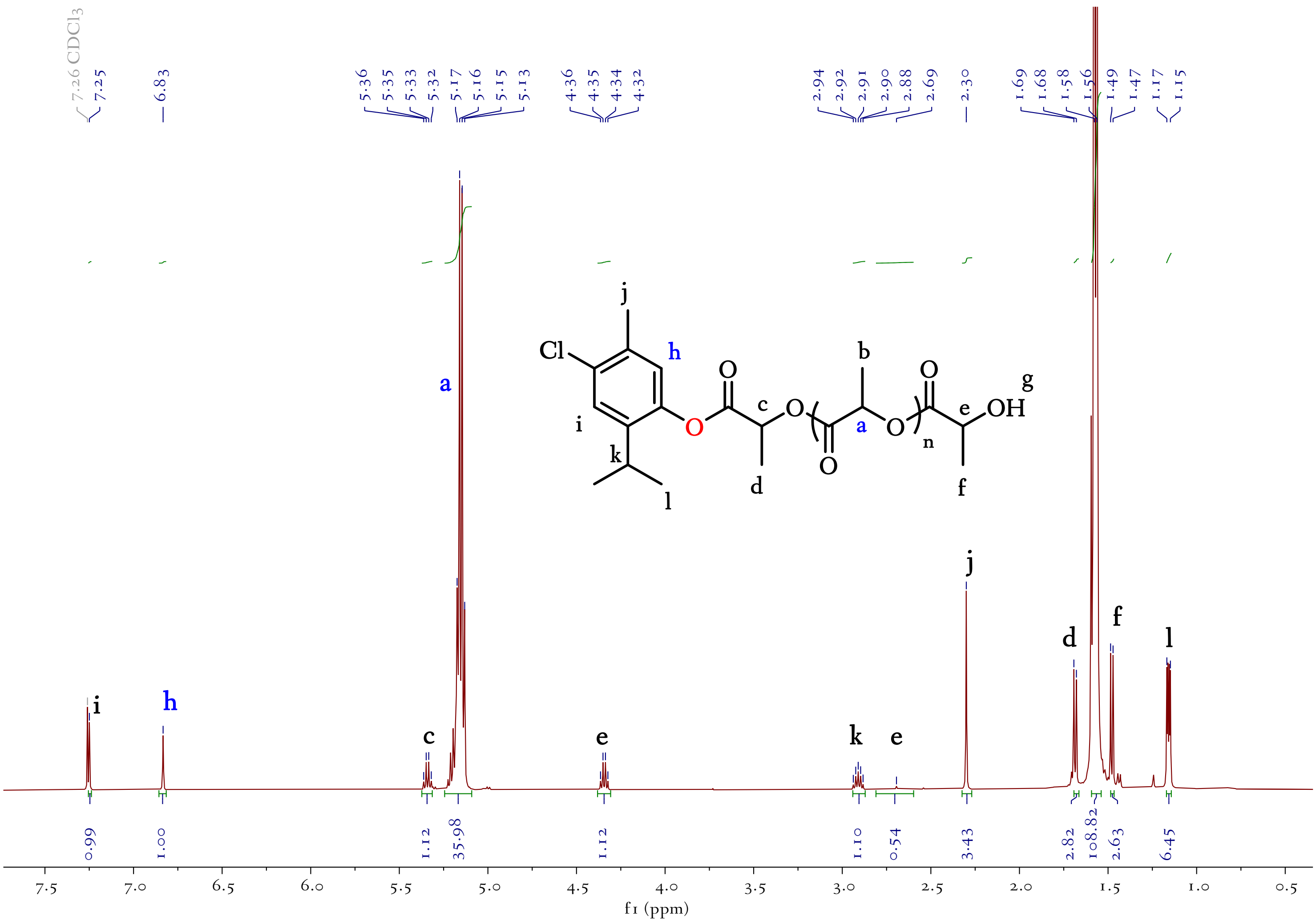

Figure S14a: ^{1}H NMR of Chlorothymol-PLA polymer, **Table 5, entry 1**.

^{1}H NMR chemical shift (δ) and coupling constant (J) values:

^{1}H NMR (400 MHz, $CDCl_3$) δ 7.25 (s, 1H), 6.83 (s, 1H), 5.34 (q, *J* = 7.1 Hz, 1H), 5.15 (q, *J* = 7.1 Hz, 36H), 4.34 (q, *J* = 6.9 Hz, 1H), 2.91 (p, *J* = 6.8 Hz, 1H), 2.69 (s, 1H), 2.30 (s, 3H), 1.69 (d, *J* = 7.1 Hz, 3H), 1.57 (d, *J* = 7.1 Hz, 108H), 1.48 (d, *J* = 7.0 Hz, 3H), 1.16 (dd, *J* = 3.3, 3.0 Hz, 6H).

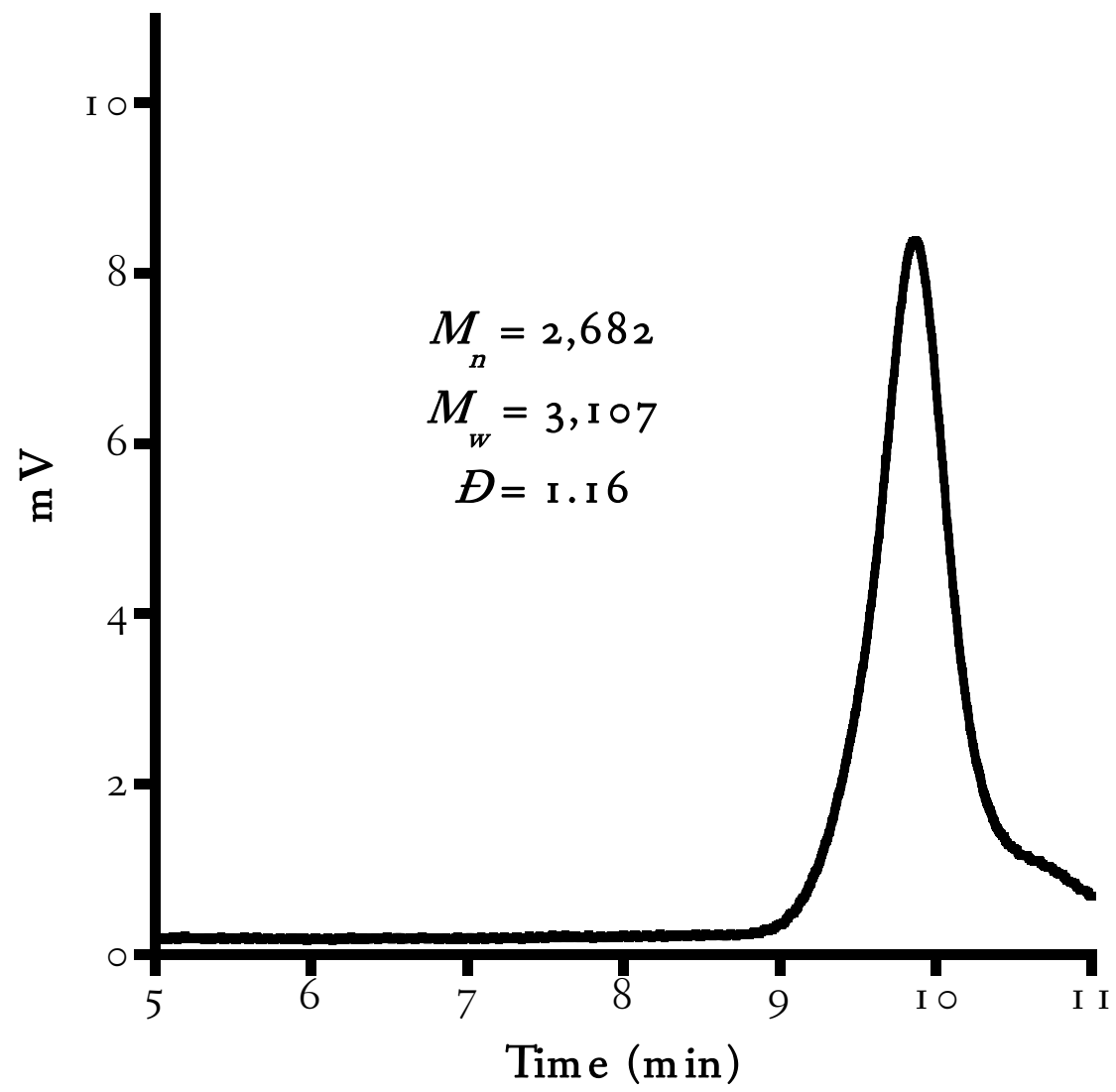

Figure S14b: GPC of Chlorothymol-PLA polymer, **Table 5, entry 1.**

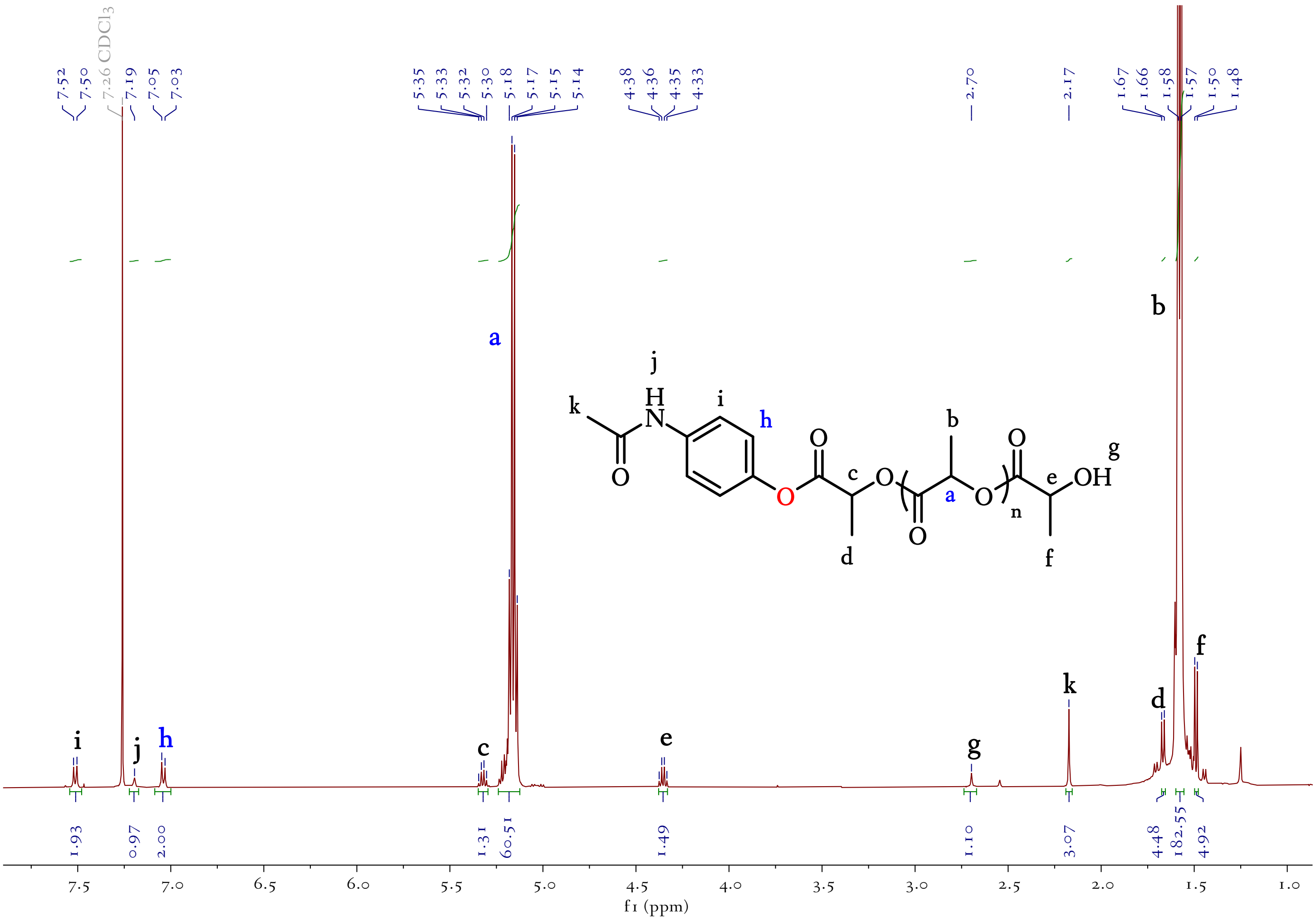

Figure S15a: ^{1}H NMR: Acetaminophen-PLA polymer, **Table 5, entry 2.**

^{1}H NMR chemical shift (δ) and coupling constant (J) values:

^{1}H NMR (400 MHz, $CDCl_3$) δ 7.51 (d, *J* = 9.2 Hz, 2H), 7.19 (s, 1H), 7.04 (d, *J* = 8.8 Hz, 2H), 5.32 (q, *J* = 7.0 Hz, 1H), 5.16 (q, *J* = 7.1 Hz, 61H), 4.35 (q, *J* = 6.9 Hz, 1H), 2.70 (s, 1H), 2.17 (s, 3H), 1.67 (d, *J* = 7.2 Hz, 3H), 1.58 (d, *J* = 7.2 Hz, 183H), 1.49 (d, *J* = 7.0 Hz, 3H).

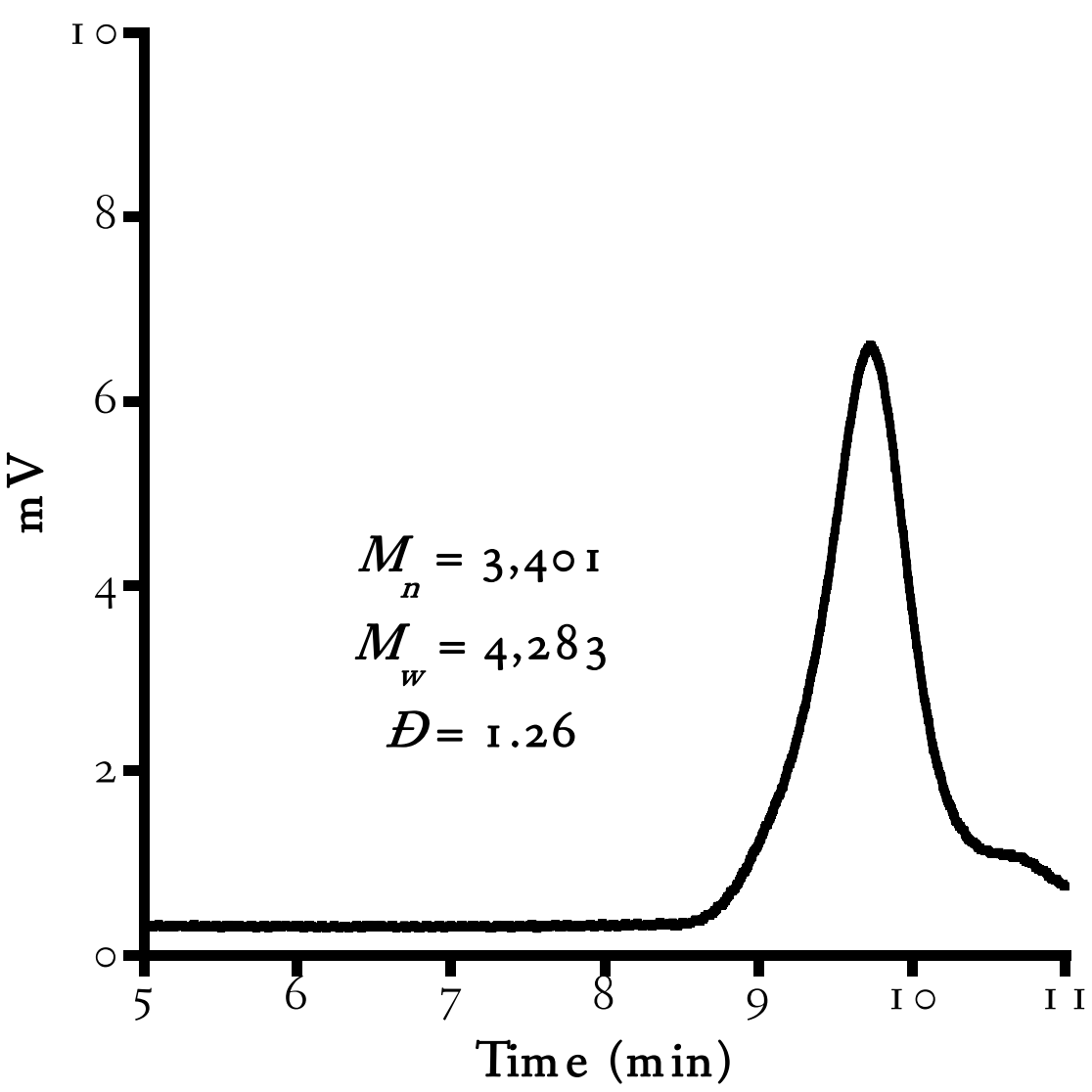

Figure S15b: GPC of Acetaminophen-PLA polymer, **Table 5, entry 2.**

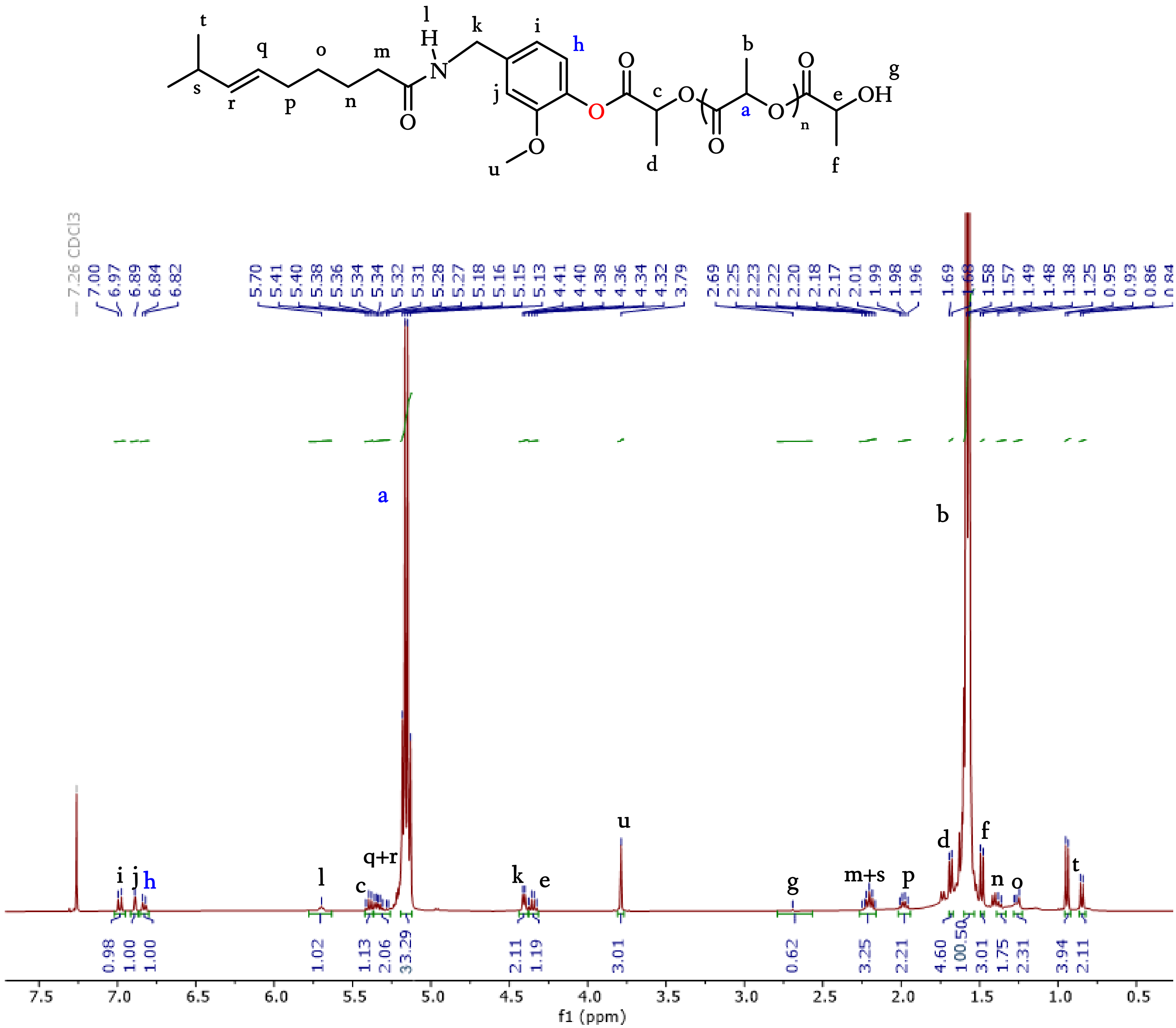

Figure S16a: ^{1}H NMR of Capsaicin -PLA polymer, **Table 5, entry 3.**

^{1}H NMR chemical shift (δ) and coupling constant (J) values:

^{1}H NMR (400 MHz, $CDCl_3$) δ 6.99 (d, *J* = 8.1 Hz, 1H), 6.89 (s, 1H), 6.83 (d, *J* = 7.8 Hz, 1H), 5.70 (s, 1H), 5.43 – 5.27 (m, 3H), 5.16 (q, *J* = 7.1 Hz, 33H), 4.41 (d, *J* = 6.0 Hz, 2H), 4.35 (q, *J* = 7.0 Hz, 1H), 3.79 (s, 3H), 2.69 (s, 1H), 2.28 – 2.15 (m, 3H), 1.98 (q, *J* = 6.9 Hz, 2H), 1.69 (d, *J* = 7.1 Hz, 3H), 1.57 (d, *J* = 7.1 Hz, 100H), 1.49 (d, *J* = 6.9 Hz, 3H), 1.45 – 1.18 (m, 4H), 0.94 (d, *J* = 6.7 Hz, 3H), 0.85 (d, *J* = 6.6 Hz, 3H).

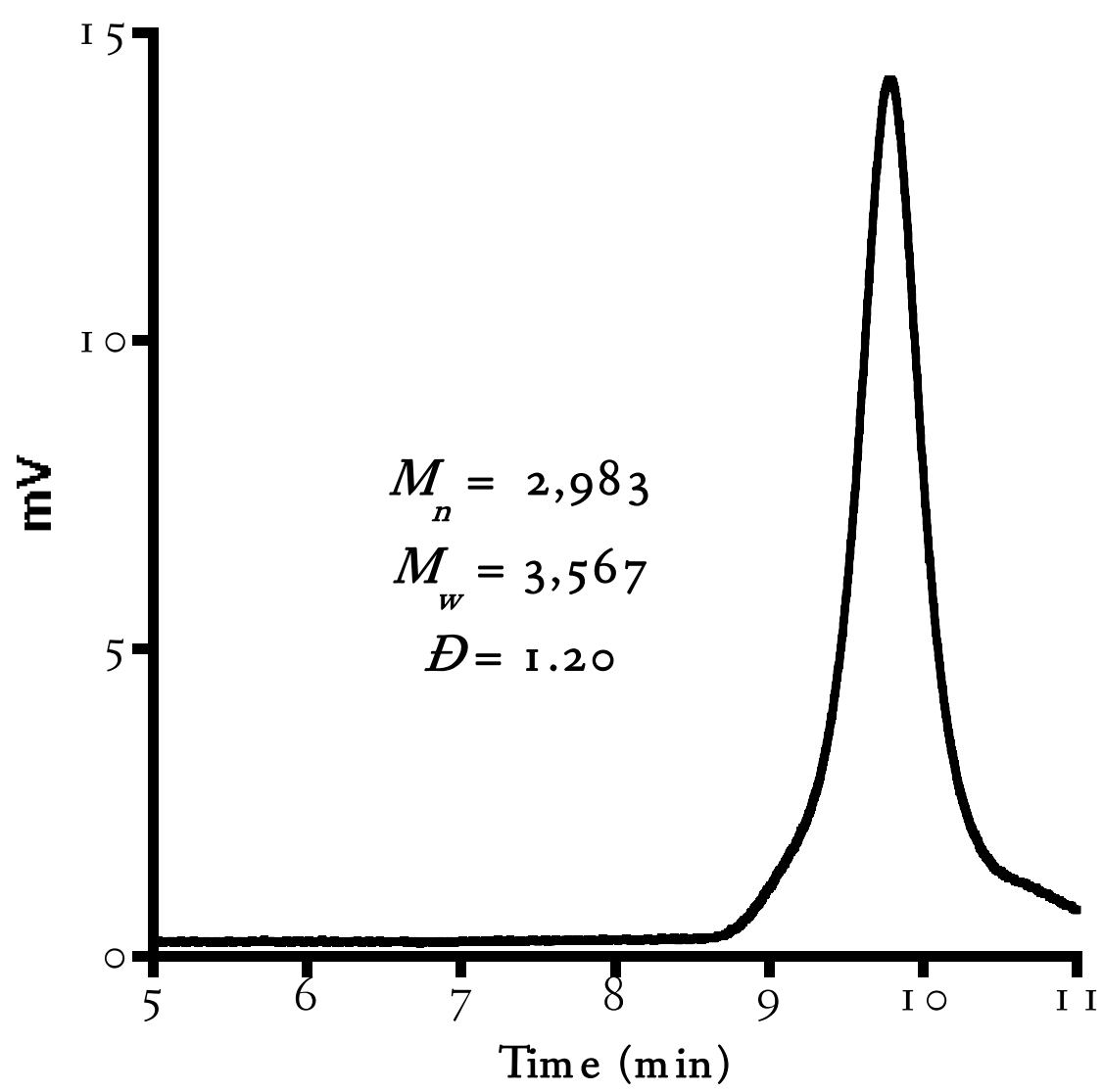

Figure S16b: GPC of Capsaicin -PLA polymer, **Table 5, entry 3.**

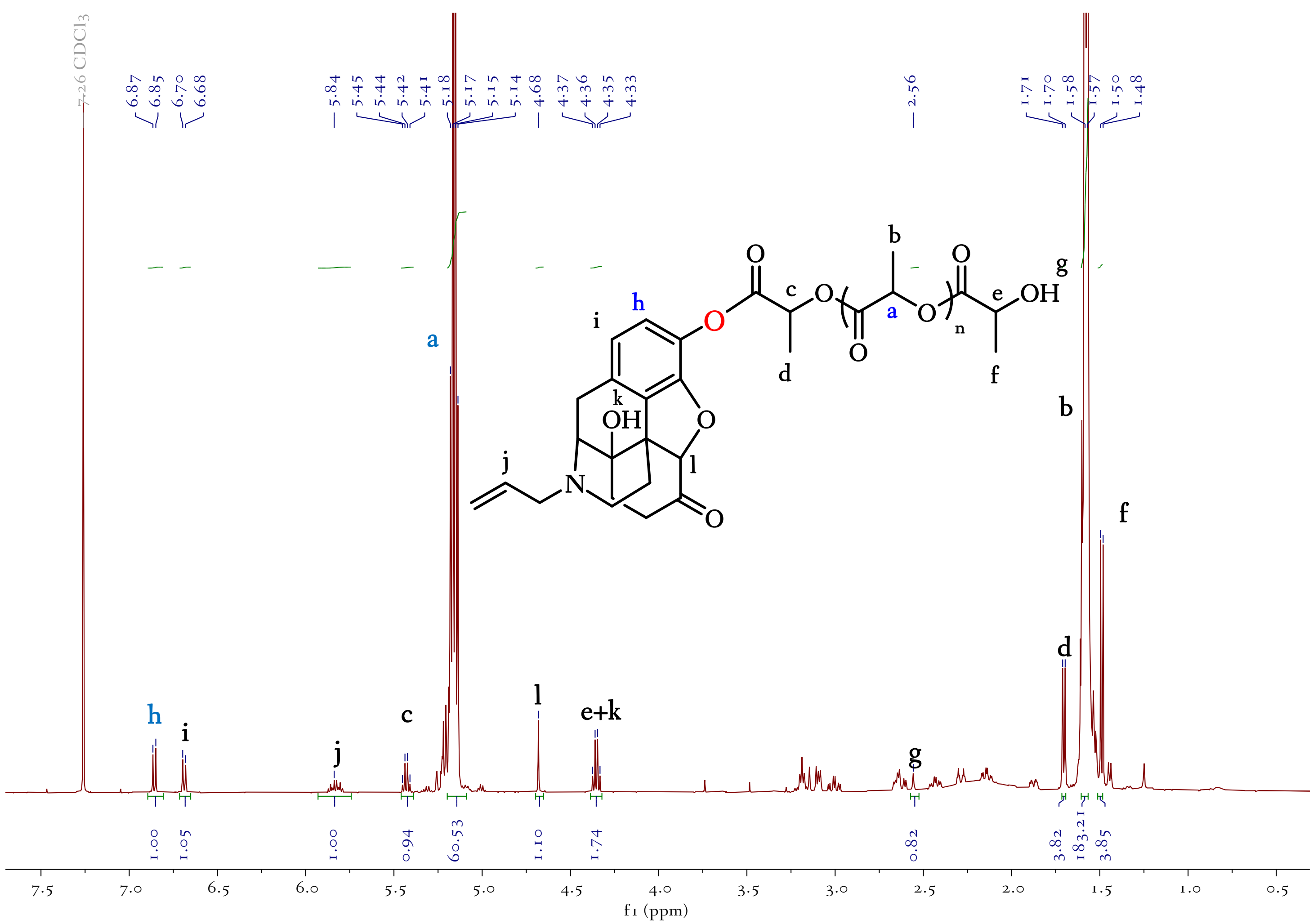

Figure S17a: ^{1}H NMR of Naloxone -PLA polymer, **Table 5, entry 4.**

^{1}H NMR chemical shift (δ) and coupling constant (J) values:

^{1}H NMR (400 MHz, $CDCl_3$) δ 6.86 (d, *J* = 8.2 Hz, 1H), 6.69 (d, *J* = 8.3 Hz, 1H), 5.84 (m, 1H), 5.43 (q, *J* = 7.0 Hz, 1H), δ 5.24 – 5.20 (m, 2H), 5.16 (q, *J* = 7.1 Hz, 61H), 4.68 (s, 1H), 4.35 (q, *J* = 7.0 Hz, 2H), 3.24 – 2.92 (m, 3H), 1.88-2.58 (m, 8H), 1.70 (d, *J* = 7.1 Hz, 3H), 1.58 (d, *J* = 7.1 Hz, 183H), 1.49 (d, *J* = 7.0 Hz, 3H), 1.34 (m, 2H).

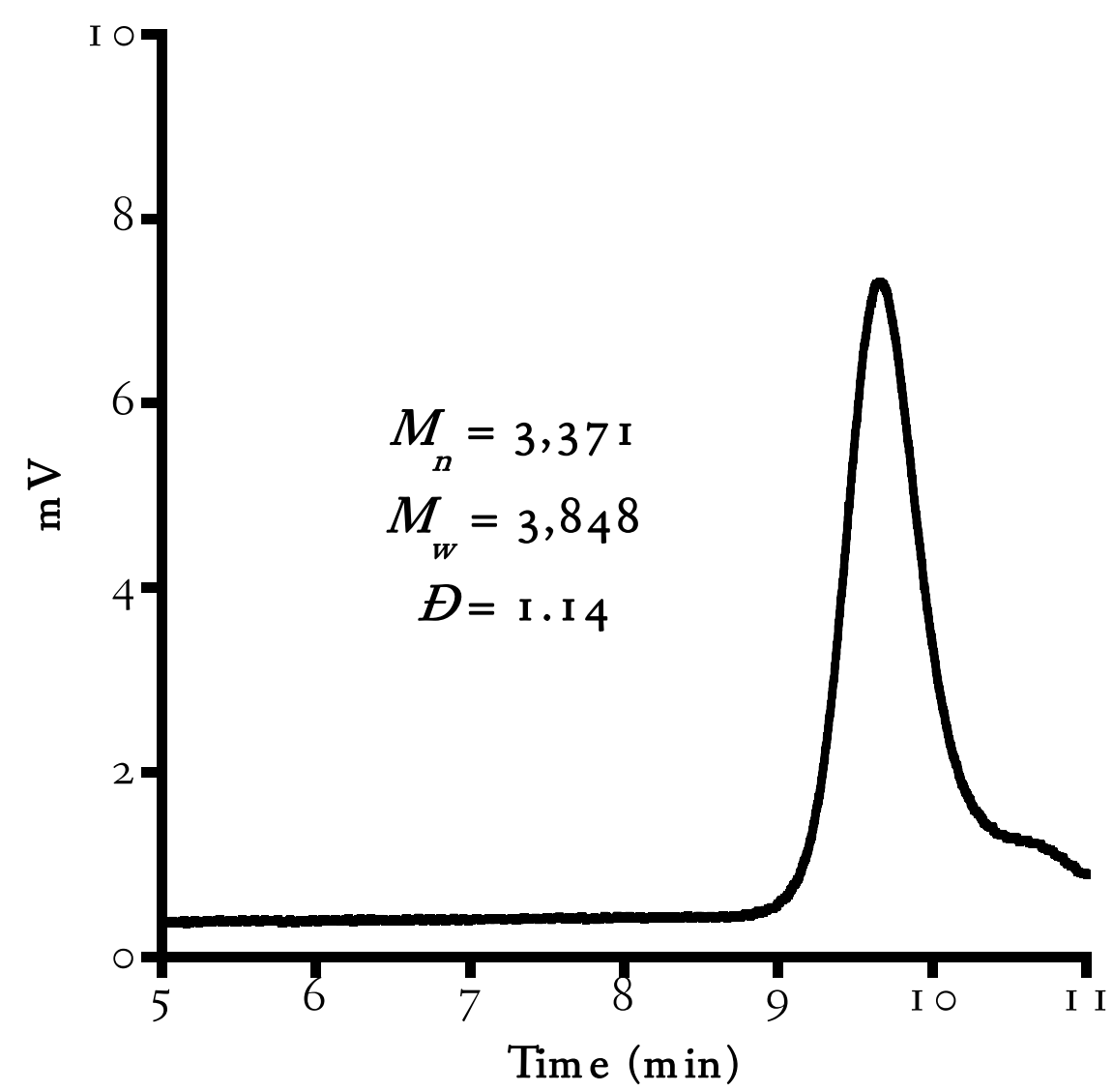

Figure S17b: GPC of Naloxone -PLA polymer, **Table 5, entry 4**

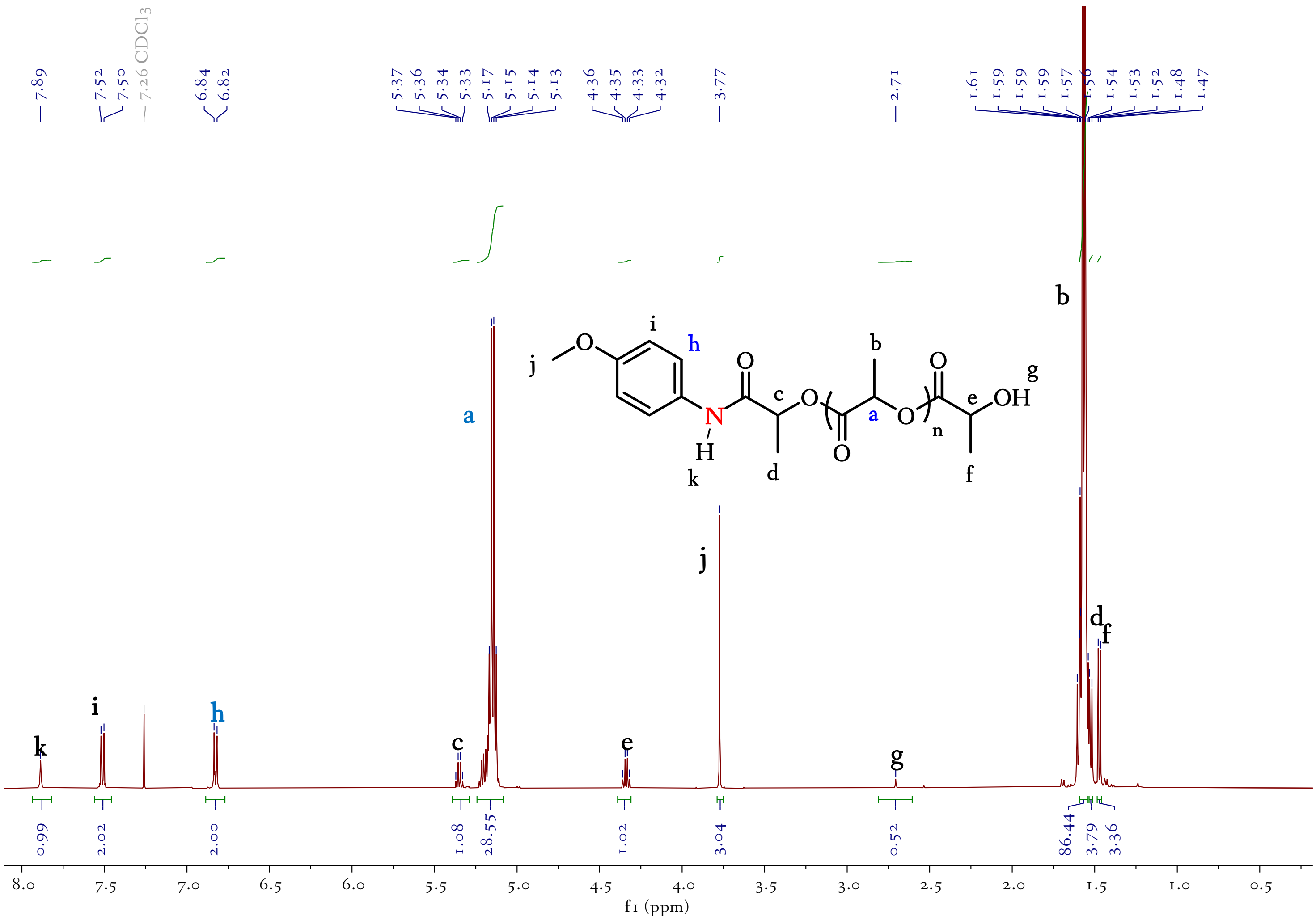

Figure S18a: ^{1}H NMR of *p*-Methoxyaniline -PLA polymer, **Table 6, entry 2.**

^{1}H NMR chemical shift (δ) and coupling constant (J) values:

^{1}H NMR (400 MHz, $CDCl_3$) δ 7.89 (s, 1H), 7.51 (d, *J* = 9.0 Hz, 2H), 6.83 (d, *J* = 9.0 Hz, 2H), 5.35 (d, *J* = 7.0 Hz, 1H), 5.15 (q, *J* = 7.0 Hz, 29H), 4.34 (d, *J* = 6.8 Hz, 1H), 3.77 (s, 4H), 2.71 (s, 1H), 1.56 (d, *J* = 7.1 Hz, 87H), 1.52 (d, *J* = 7.1 Hz, 3H), 1.47 (d, *J* = 7.0 Hz, 3H).

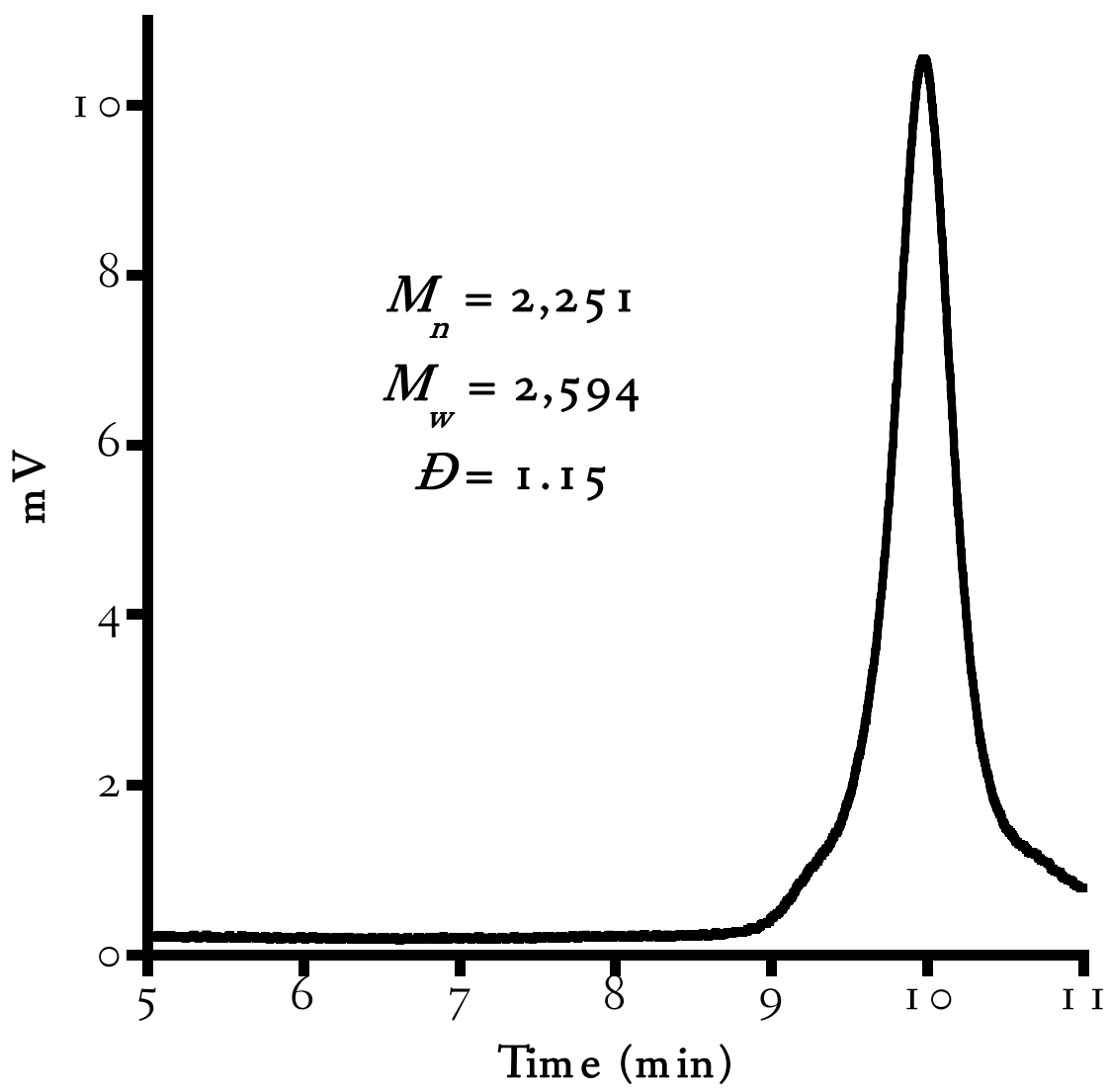

Figure S18b: GPC of *p*-Methoxyaniline -PLA polymer, **Table 6, entry 2.**

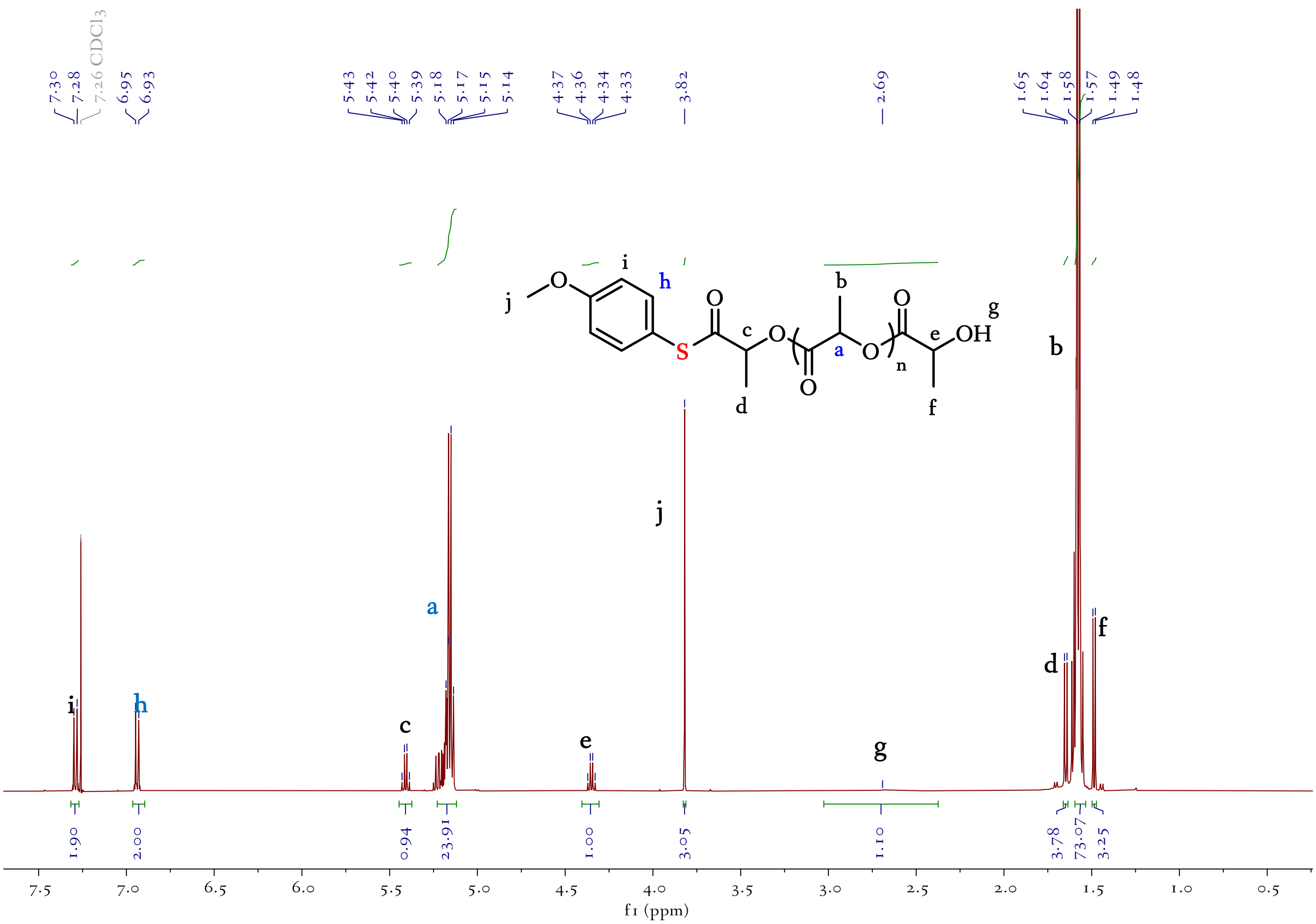

Figure S19a: ¹H NMR of *p*-Methoxythiophenol -PLA polymer, **Table 6, entry 3.**

¹H NMR chemical shift (δ) and coupling constant (J) values:

¹H NMR (400 MHz, $CDCl_3$) δ 7.29 (d, *J* = 9.0 Hz, 2H), 6.94 (d, *J* = 9.0 Hz, 2H), 5.41 (q, *J* = 7.0 Hz, 1H), 5.19 – 5.11 (m, 24H), 4.35 (q, *J* = 6.9 Hz, 1H), 3.82 (s, 3H), 2.69 (s, 1H), 1.65 (d, *J* = 7.1 Hz, 3H), 1.58 (d, *J* = 7.2 Hz, 72H), 1.49 (d, *J* = 7.0 Hz, 3H).

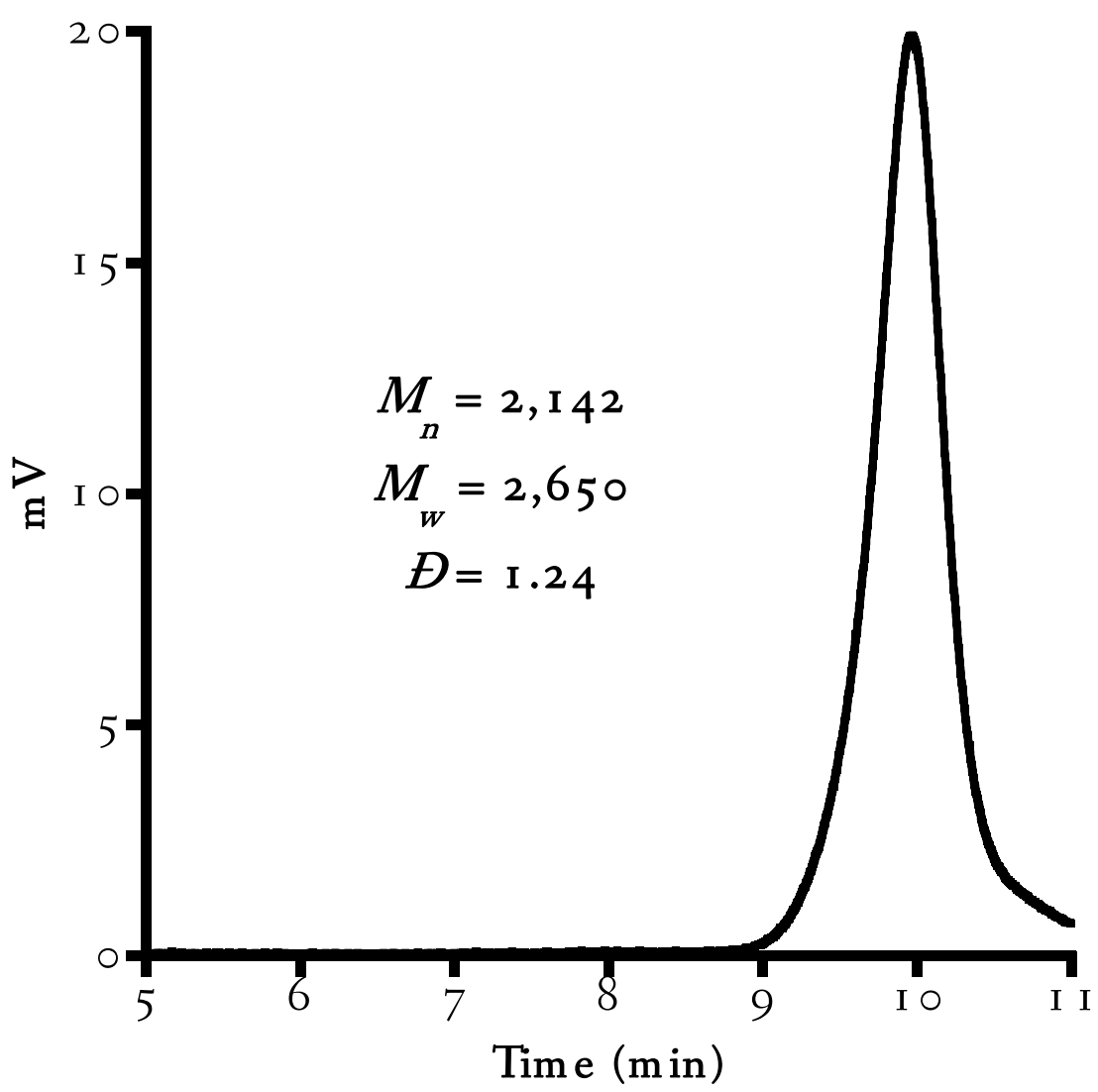

Figure S19b: GPC of *p*-Methoxythiophenol -PLA polymer, **Table 6, entry 3.**

References

1. J. M. Pérez, C. Ruiz and I. Fernández, Synthesis of a biodegradable PLA: NMR signal deconvolution and end-group analysis, *J. Chem. Educ.*, 2022, ***99***, 1000–1007, DOI: 10.1021/acs.jchemed.1c00824.

2. M. A. Ebqa'ai, S. B. Kasthuri Dias, A. J. Kassick, S. Averick and T. L. Nelson, Naloxone-initiated mechanochemical synthesis of poly(lactic acid), *RSC Mechanochem.*, 2025, ***2***, 25–29, DOI: 10.1039/D4MR00046C.
